# Honeysuckle (*Lonicera japonica*) and Huangqi (*Astragalus membranaceus*) Suppress SARS-CoV-2 Entry and COVID-19 Related Cytokine Storm *in Vitro*

**DOI:** 10.3389/fphar.2021.765553

**Published:** 2022-03-25

**Authors:** Yuan-Chieh Yeh, Ly Hien Doan, Zi-Yi Huang, Li-Wei Chu, Tzu-Hau Shi, Ying-Ray Lee, Cheng-Tao Wu, Chao-Hsiung Lin, Shu-Tuan Chiang, Hui-Kang Liu, Tsung-Hsien Chuang, Yueh-Hsin Ping, Hsiao-Sheng Liu, Chi-Ying F. Huang

**Affiliations:** ^1^ Department of Traditional Chinese Medicine, Chang Gung Memorial Hospital, Keelung, Taiwan; ^2^ Program in Molecular Medicine, College of Life Sciences, National Yang Ming Chiao Tung University, Taipei, Taiwan; ^3^ Institute of Biopharmaceutical Sciences, College of Pharmaceutical Sciences, National Yang Ming Chiao Tung University, Taipei, Taiwan; ^4^ Institute of Biotechnology, Vietnam Academy of Science and Technology, Hanoi, Vietnam; ^5^ ASUS Intelligent Cloud Services, Taipei, Taiwan; ^6^ Department and Institute of Pharmacology, College of Medicine, National Yang Ming Chiao Tung University, Taipei, Taiwan; ^7^ Department of Life Sciences and Institute of Genome Sciences, College of Life Sciences, National Yang Ming Chiao Tung University, Taipei, Taiwan; ^8^ Department of Medical Research, Ditmanson Medical Foundation Chia-Yi Christian Hospital, Chiayi, Taiwan; ^9^ Department of Microbiology and Immunology, School of Medicine, College of Medicine, Kaohsiung Medical University, Kaohsiung, Taiwan; ^10^ Division of Big Data, Phalanx Biotech Group, Hsinchu, Taiwan; ^11^ Aging and Health Research Center, National Yang Ming Chiao Tung University, Taipei, Taiwan; ^12^ Chuang Song Zong Pharmaceutical Co., Ltd. Ligang Plant, Pingtung, Taiwan; ^13^ National Research Institute of Chinese Medicine (NRICM), Ministry of Health and Welfare, Taipei, Taiwan; ^14^ Ph. D. Program in the Clinical Drug Development of Herbal Medicine, Taipei Medical University, Taipei, Taiwan; ^15^ Immunology Research Center, National Health Research Institutes, Miaoli, Taiwan; ^16^ Program in Environmental and Occupational Medicine, Kaohsiung Medical University, Kaohsiung, Taiwan; ^17^ Institute of Biophotonics, College of Biomedical Science and Engineering, National Yang Ming Chiao Tung University, Taipei, Taiwan; ^18^ Department of Microbiology and Immunology, College of Medicine, National Cheng Kung University, Tainan, Taiwan; ^19^ Center for Cancer Research, College of Medicine, Kaohsiung Medical University, Kaohsiung, Taiwan; ^20^ M.Sc. Program in Tropical Medicine, College of Medicine, Kaohsiung Medical University, Kaohsiung, Taiwan; ^21^ Institute of Clinical Medicine, College of Medicine, National Yang Ming Chiao Tung, Taipei, Taiwan; ^22^ Department of Biotechnology and Laboratory Science in Medicine, School of Biomedical Science and Engineering, National Yang Ming Chiao Tung, Taipei, Taiwan; ^23^ Department of Biochemistry, School of Medicine, Kaohsiung Medical University, Kaohsiung, Taiwan

**Keywords:** honeysuckle, Huangqi, COVID-19, SARS-CoV-2, microRNA, let-7a, miR-148b, mir-146a

## Abstract

COVID-19 is threatening human health worldwide but no effective treatment currently exists for this disease. Current therapeutic strategies focus on the inhibition of viral replication or using anti-inflammatory/immunomodulatory compounds to improve host immunity, but not both. Traditional Chinese medicine (TCM) compounds could be promising candidates due to their safety and minimal toxicity. In this study, we have developed a novel *in silico* bioinformatics workflow that integrates multiple databases to predict the use of honeysuckle (*Lonicera japonica*) and Huangqi (*Astragalus membranaceus*) as potential anti-SARS-CoV-2 agents. Using extracts from honeysuckle and Huangqi, these two herbs upregulated a group of microRNAs including *let-7a*, *miR-148b*, and *miR-146a*, which are critical to reduce the pathogenesis of SARS-CoV-2. Moreover, these herbs suppressed pro-inflammatory cytokines including IL-6 or TNF-α, which were both identified in the cytokine storm of acute respiratory distress syndrome, a major cause of COVID-19 death. Furthermore, both herbs partially inhibited the fusion of SARS-CoV-2 spike protein-transfected BHK-21 cells with the human lung cancer cell line Calu-3 that was expressing ACE2 receptors. These herbs inhibited SARS-CoV-2 M^pro^ activity, thereby alleviating viral entry as well as replication. In conclusion, our findings demonstrate that honeysuckle and Huangqi have the potential to be used as an inhibitor of SARS-CoV-2 virus entry that warrants further *in vivo* analysis and functional assessment of miRNAs to confirm their clinical importance. This fast-screening platform can also be applied to other drug discovery studies for other infectious diseases.

## Introduction

The rapid spread of SARS-CoV-2 causing the coronavirus disease 2019 (COVID-19) pandemic since the late 2019 has a tremendous impact on global public health systems (Wang et al., 2020). The mortality rate of COVID-19 is 2.3% (https://coronavirus.jhu.edu/map.html), and the transmission rate is increasing due to the more lethal SARS-CoV-2 variants (Guo et al., 2020; Davies et al., 2021a; Davies et al., 2021b). Therefore, new therapeutic drugs are urgently needed to prevent medical support overload.

Drug development for COVID-19 are focusing on anti-viral drugs for the viral phase and anti-inflammatory/immunomodulatory drugs for the inflammatory phase (Meganck and Baric, 2021). Many registered clinical trials are on-going (Kupferschmidt and Cohen, 2020) and several drugs are approved by FDA for Emergency Use Authorization (EUA), such as remdesivir, casirivimab, imdevimab, and so on. However, most anti-viral agents target a limited number of pathways that may not be related to the pathophysiology of SARS-CoV-2 infection; also, these agents may cause adverse effects (Lucas et al., 2001; Stokkermans and Trichonas, 2020; Catalano et al., 2021; Meganck and Baric, 2021). In contrast, traditional Chinese medicine (TCM) drugs may be excellent anti-viral drug candidates since most of them have minimal toxicity and mild side effects (Lau et al., 2005; Poon et al., 2006; Ding et al., 2017a). Many complex TCM formulas are in clinical testing for COVID-19 based on experience, but most of them lack of rationale or systems biology-based analysis on molecular mechanisms (Yang et al., 2020).

Current research hotspots for anti-SARS-CoV-2 targets include spike protein, angiotensin converting enzyme 2 (ACE2), transmembrane serine protease 2 (TMPRSS2), protease, endosome, and RNA dependent RNA replication (RdRp) (Hoffmann et al., 2020; Leng et al., 2020; Sumon et al., 2020). Therefore, we aimed to search for TCM drugs to block the binding of spike protein to ACE2 receptor and their syncytia formation, as well as to inhibit viral replication *via* SARS-CoV-2 M^pro^. M^pro^, a protease residing in polyproteins 1a and 1ab (pp1a and pp1ab) that are composed of multiple non-structural proteins, is essential for viral replication. During SARS-CoV-2 replication, M^pro^ proteolytically cleaves the non-structural proteins required for viral replication; thus, M^pro^ is a promising target for therapeutic intervention against COVID-19 (V'Kovski et al., 2021). On the other hand, acute respiratory distress syndrome (ARDS) is one of complications due to a consequence of virus-induced uncontrolled cytokine storm (Wu Y et al., 2020; Liu et al., 2020). Most COVID-19 patients with ARDS are associated with elevated levels of various cytokines, including interleukin (IL)-2, IL-6, IL-7, interferon-γ inducible protein 10 (CXCL10), granulocyte colony-stimulating factor (G-CSF), and tumor necrosis factor-α (TNF-α) (Huang et al., 2020).

Our goal was to provide a list of potential anti-SARS-CoV-2 TCM drugs covering a wide range of pharmacologic functions that we could integrate into clinical practice. However, it is challenging to comprehensively screen for anti-SARS-CoV-2 TCMs due to the diversity and complexity of TCM drugs. Here, we addressed these challenges by performing a systematic big data analysis that integrated several databases to connect small-molecular’ targets and TCM-associated targets. Using our bioinformatics workflow, we identified two common Chinese herbs, honeysuckle (*Lonicera japonica*) and Huangqi (*Astragalus membranaceus*), that display similar anti-SARS-CoV-2 characteristics. The flower or dry bud of honeysuckle is traditionally used as an anti-inflammatory herb, and it is efficacious for treating various viral infections, such as hepatitis B virus, adenovirus, influenza A virus, dengue virus, enterovirus, and respiratory syncytial virus (Yu et al., 2013; Ding et al., 2017b; Lee et al., 2017; Li et al., 2017; Ma et al., 2017; Ge et al., 2019; Li J et al., 2020; Lee et al., 2021). Honeysuckle is safe to be used as food and medicine because it has been used for thousand years of practicing TCM (Li RJ et al., 2020). Moreover, Huangqi is rich in the anti-viral immunomodulatory compound *Astragalus* polysaccharide (APS) (Shi et al., 2014; Xue et al., 2015; Wang et al., 2016; Zheng et al., 2020); thus, it may be effective against SARS-CoV-2 infections.

MicroRNAs (miRNAs), small non-coding RNAs, can attach to target mRNAs, resulting in the degradation or translational inhibition of corresponding mRNAs (Chandan et al., 2019). As pathogens generally exploit miRNAs for their survival and replication in the host body, the modification of miRNA expression has been investigated widely in infectious diseases (Acuña et al., 2020). Our team has shown that honeysuckle-induced host *let-7a* can inhibit dengue virus and enterovirus 71 replications by targeting the specific regions of the viral genomes (Lee et al., 2017; Lee et al., 2021). Furthermore, both *let-7a* and *miR-148b* were also predicted to target SARS-CoV-2 genome sequences simultaneously at multiple regions (Lee et al., 2021). *Let-7a* can not only inhibit viral replication but also attenuate cytokine storm that leads to ARDS, a leading cause of death among COVID-19 patients (Jiang et al., 2019; Jin et al., 2019; Li RJ et al., 2020). In addition, previous clinical data revealed that IL-6 and TNF-α were the most critical cytokines detected in patients with severe COVID-19 symptoms (Han et al., 2020; Mandel et al., 2020; Choudhary et al., 2021). The low expression level of *miR-146a* in the sera of COVID-19 patients was reported to correlate with unfavorable consequences (Sheedy and O'Neill, 2008; Sabbatinelli et al., 2021). The secretion of IL-6 is induced by NF-κB signaling pathway, which is negatively regulated by *miR-146* (Sheedy and O'Neill, 2008). NF-κB enhances the synthesis of several inflammation-related proteins, such as TNF-α. Therefore, the interaction among NF-κB, TNF-α, and IL-6 forms a positive feedback loop, contributing to deadly cytokine storms.

Herein we established a novel *in silico* approach to construct a comprehensive map of TCM drugs that might have potential for COVID-19 treatment. Moreover, we demonstrated empirically the therapeutic potential of honeysuckle and Huangqi that could inhibit the viral infection process by blocking the binding of spike protein-ACE2, suppressing SARS-CoV-2 M^pro^ and inflammatory phase by targeting cytokines for the prevention and treatment of COVID-19.

## Materials and Methods

### TCM Drugs Prediction Workflow Across Multiple Databases

**FIGURE 1:**
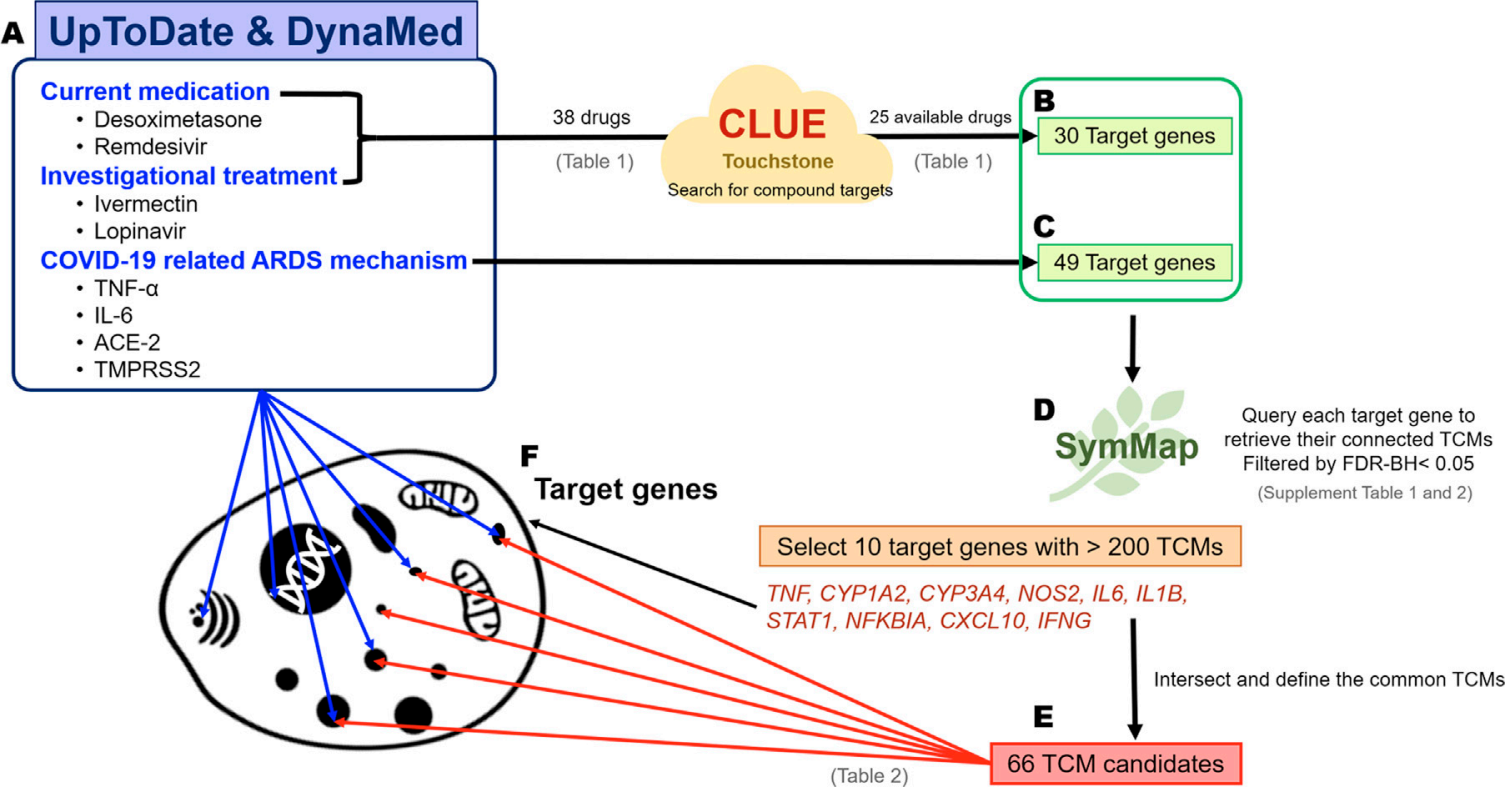
The schematic for predicting TCM candidates and the workflow of the analysis of their mechanisms of action. Identification of potential TCM candidates for COVID-19 treatment. The target genes indicate the biological function affected by the drug treatment and viral mechanisms. Therefore, small-molecule reagents, viral mechanisms, and the TCM candidates can be linked through the common shared target genes. The flow chart describes the steps in the bioinformatics analysis. **(A)** The 38 well-known small-molecule reagents for COVID-19 treatments were obtained from UpToDate and DynaMed, and 25 out of 38 drugs had the corresponding drug targets via CLUE (TOUCHSTONE). **(B)** A total of 30 target genes could be obtained from 25 drugs. **(C)** Forty-nine target genes were identified by reviewing the COVID-19-related ARDS mechanisms and were included along with the abovementioned 30 target genes for further analysis. Two target genes, TNF and TLR7, were present in both target gene groups. **(D)** The target above genes were queried separately using SymMap to retrieve their connected TCMs. The ten most frequent target genes were connected to more than 200 TCMs. **(E)** As a result, 66 TCM candidates shared common connections with all of these 10 target genes. **(F)** The main goal of our big data analysis was to link the small-molecule agents and TCM via biological target genes.

Known viral mechanisms, drug candidates, and investigational treatments of COVID-19 were collected from the online evidence-based retrieval databases UpToDate (https://www.uptodate.com/) and DynaMed (https://www.dynamed.com/) (Weng et al., 2014), and analyzed for the current and potential treatments (Figure 1). The target genes involved in known viral mechanisms were also retrieved, whereas the target genes of current medications and investigational drugs for COVID-19 were obtained from CLUE (TOUCHSTONE) (https://clue.io/) (Subramanian et al., 2017). Both target gene sets were used to query the SymMap database (https://www.symmap.org), which offers six categories of information, including herbal name, genetic target, ingredient, modern medicine symptom, TCM symptom, and disease target. We can then obtain many herbs, which were ranked by the pairwise relationships among the TCM candidates and the viral target genes of interest (our target genes). Briefly, target genes were used to query separately to SymMap with FDR-BH <0.05 to acquire connected TCM drugs. Because each target gene might link to many TCM drugs, intersection of all TCM candidates was then performed to reduce the number of TCM candidates.

### Mechanism Analysis of TCM Drugs and miRNAs *via* Big Data Analysis

We obtained the TCM target genes with FDR-BH < 0.01 from SymMap; *let-7a-5p*, *miR-148b-5p*, and *miR-146a-5p*-targeted genes were from miRDB (http://mirdb.org/) (Chen and Wang, 2020). The biological mechanisms of the TCM candidates and miRNAs of interest were analyzed through ConsensusPathDB (CPDB) (http://cpdb.molgen.mpg.de/) (Zhang and Guo, 2020), a free public online software, and Ingenuity Pathways Analysis (IPA) (Jia et al., 2021), a commercial platform. Both databases collected the comprehensive pathway information across diversities resources. Querying the set of the identified target genes in CPDB and IPA revealed potential pathways. The cutoff *p*-value for CPDB and IPA was 0.05. Furthermore, TCM candidates could be predicted to associate with specific miRNAs through g:Profiler (https://biit.cs.ut.ee/gprofiler/), a web-based functional enrichment analytical software (Raudvere et al., 2019). This method had been reported in our previous study (Lee et al., 2021). In this analysis, the statistical domain scope of g:Profiler was set as “all known genes,” and the significance threshold was set as FDR-BH <0.05.

For the connection of candidate herbs and COVID-19, the COVID-19 disease signature was adapted from gene expression profile of GSE147507 (Blanco-Melo et al., 2020). Calu-3, a human lung cancer cell line, was infected by SARS-CoV-2. The fold change level of its gene expression was detected to analyze the pathological mechanism and define potential targets of SARS-CoV-2.

### Honeysuckle and Huangqi Preparations

The extracts of honeysuckle (*Lonicera japonica*) were provided by Chuang Song Zong Pharmaceutical Co. Ltd., Ligang Plant, Pingtung, Taiwan. The aqueous extract of honeysuckle (honeysuckle-H_2_O) was prepared by boiling 50 g of dried honeysuckle flower buds in 500 ml of double-distilled water and refluxing for 90 min twice. After filtering followed by vacuum concentration, a total of 20.92 g honeysuckle-H_2_O extractives was collected. An ethanol extract of honeysuckle (honeysuckle-EtOH) was prepared by mixing 50 g of dried honeysuckle flower buds with 500 ml of 95% ethanol and heating to reflux for 90 min twice. A total of 9.78 g of honeysuckle-EtOH extractives was obtained after filtering, followed by vacuum concentration. HPLC fingerprints for honeysuckle-H_2_O and honeysuckle-EtOH extracts were shown in Supplementary Figures S1, S2, respectively.

The extract of Huangqi was prepared as APS and APS-L, respectively. *Astragalus* polysaccharides was extracted from the *Astragalus membranaceus* (AM) root (PhytoHealth Co. Ltd., Taipei, Taiwan) *via* a series of refining processes. First, the APS extract was prepared by hot water extraction of AM, followed by alcoholic precipitation of the condensed water-soluble extracts. The alcohol-precipitated slurry was further spray-dried to remove the residual solvent to obtain APS in the form of a pale-yellow powder. The remaining supernatant was vacuum concentrated to a dark red or brown-colored paste (APS-L). A Certificate of Analysis (CoA) was obtained for APS and APS-L to ensure batch consistency of the carbohydrate contents, pH, appearance, loss on drying, and total residual alcohol content. The CoA also complied with the standards of no heavy metals, plasticizers, or microbes, as confirmed by microbial counts, total combined yeasts/molds, and the presence of *Escherichia coli* and *Salmonella* spp.

### Cell Culture

THP-1, a non-adherent human monocytic cell line derived from an acute monocytic leukemia patient (ATCC TIB-202), purchased from Bioresource Collection and Research Center (BCRC), Taiwan were cultured in Roswell Park Memorial Institute (RPMI) (Gibco) 1640 supplemented with 10 mM HEPES, 1% penicillin-streptomycin (PS) and 50 µM 2-ME, and sustained in Petri dish in a 5% carbon dioxide-humidified atmosphere at 37°C. Cell were continuously passaged after 3–4 days.

BEAS2B cells, derived from the normal bronchial epithelium of a non-cancerous human were used to screen for biological agents affecting infection mechanisms in the respiratory tract (Shukla et al., 2016), were grown in adherent cultures, maintained at 37°C, 5% CO_2_, in RPMI medium supplemented with 10% fetal bovine serum (FBS: Invitrogen), 1% PSA and 1% nonessential amino acid. 2 mM l-glutamate (Invitrogen) also added to RPMI medium. BEAS2B was cultured in. The cell cultures were passaged by trypsinization every 3–4 days.

### qRT-PCR Analysis

We determined whether candidate drugs could induce the expression of *let-7a*, *miR-148b*, or *miR-146a*. First, 1 × 10^6^ BEAS2B cells were seeded in a 10-cm dish 24 h before the drug treatment. The cells were collected 24 h after the drug treatment. TRIzol^®^ reagent was used for total RNA extraction, and RNA samples were stored at −80°C. The miRNA levels of *let-7a*, *miR-148b*, and *miR-146a* expression were quantified using quantitative reverse transcription PCR (qRT-PCR) with U54 as the internal control. Real time-PCR primers for amplification were used, including forward sequences specific for *hsa-let-7a-5p* (5′-GCC​TGA​GGT​AGT​AGG​TTG​TAT​AGT​TA-3′), *hsa-miR148b-5p* (5′-AAG​UUC​UGU​UAU​ACA​CUC​AGG​C-3′)*, has-miR-146a* (5′-UGA​GAA​CUG​AAU​UCC​AUG​GGU​U-3′), and U54 (homo) (5′-GGT​ACC​TAT​TGT​GTT​GAG​TAA​CGG​TGA-3′). qRT-PCR was performed using Phalanx miRNA OneArray^®^ Profiling (Phalanx Biotech Group).

### Cytokine Assays

THP-1 cells, differentiated by treatment with 50 ng/ml phorbol 12-myristate 13-acetate (PMA) (SIGMA; P1585) for 24 h, were used as the cell model. In addition, lipopolysaccharide (LPS) (SIGMA; L2654) was used as a stimulator to mimic the inflammatory condition and the treatment of LPS 100 ng/ml alone on differentiated THP-1 cells was considered as control. The cells were treated with the drug candidates with or without the presence of LPS and incubated at 37°C for 6 or 24 h. In addition, in mimicking SARS-CoV-2-induced cytokine storm assay, THP-1 cells were seeded with 5 × 10^5^ cells per well in 24-well plate and differentiated by PMA 50 ng/ml for 24 h, followed by PMA-free medium incubation for another 24 h. Honeysuckle (EtOH) 200 and 1,000 μg/ml were pre-treated for 2 h in combined treatment of honeysuckle and stimulators [50 ng/ml of lipopolysaccharide (LPS) (SIGMA, L2654); 11.2 nM of SARS-CoV-2 spike (ECD) protein (His tag) (Genetex, GTX02774-pro); 5 µM of R848 (Invivogen)]. Next, all medium was removed. Differentiated THP-1 cells were then treated by honeysuckle 200 or 1,000 μg/ml with or without the presence of stimulators for 24 h. After the incubation time, the cell medium was then collected and stored at −20°C. The cytokines levels induced by the treatments were determined using an enzyme-linked immunosorbent assay (ELISA) assay. The supernatants were analyzed on Nunc MaxiSorp^®^ flat-bottom 96-well plates (Invitrogen, ThermoFisher; 442402) separately for the cytokines using a human uncoated ELISA kit (Invitrogen, ThermoFisher). The optical density (OD) values were measured by an Infinite 200Pro OD reader using Tecan i-control at the wavelength of 450 and 570 nm.

### Sulforhodamine B Colorimetric Assay

BEAS2B cells were seeded at 2,000 cells per well for 16–20 h and treated with drug candidates at different concentrations for 24 h. Next, the medium was discarded, and the cells were fixed with cold 10% trichloroacetic acid (w/v) (Sigma-Aldrich) at 4°C for 1 h. Then, the plates were washed twice with water, stained with 100 μL of 0.1% (w/v, in 1% acetic acid) SRB solution per well at room temperature for 1 h, and washed twice with 1% acetic acid (AVANTOR). After air-drying, 100 μL of 20 mM Tris-base was added to each well, and the absorbance was read at the OD of 540 nm.

### Enzymatic Assays

The activity of SARS-CoV-2 M^pro^ was determined by its cleavage of a fluorogenic peptide substrate (Abz-TSAVLQSGFRK-Dnp) in 20 mM phosphate-buffered saline (PBS), pH 7.6, at 30°C for 3 min. The quencher, dinitrophenyl (Dnp), was released from fluorophore aminobenzoyl (Abz) after cleavage by M^pro^, causing fluorescence emission at 423 nm and enabling detection at the excitation wavelength of 321 nm using a luminescence spectrometer (PerkinElmer LS50B) (Shi et al., 2020). The peptide substrate concentration in the reaction ranged from 2 to 40 µM in PBS buffer with the concentration of M^pro^ at 0.12 µM. The kinetics parameters of M^pro^ vs. different substrate concentrations were determined by plotting with the classical Michaelis–Menten equation using Prism 6 (GraphPad).

### Inhibition of M^pro^ Activity and Determination of the Half Maximal Inhibitory Concentration

The inhibitory ability of M^pro^ activity of candidate drugs were determined. For example, an inhibitor, honeysuckle-EtOH, was first incubated with a fluorogenic peptide substrate in PBS at 30°C for 3 min. Then, 0.12 µM M^pro^ was added, and the reaction was equilibrated at 30°C for 3 min. The IC_50_ value was obtained using the following equation:

$$v=\frac{v_{0}}{\left(1+IC_{50}^{n}\right)/{\left[I\right]}^{n}}$$

where *v* is the velocity of cleavage at different concentrations of the inhibitor [I] and $v_{0}$ was the initial velocity without the inhibitor, whereas  n was the Hill constant.

### The Cell–Cell Fusion Assay

Human lung cancer Calu-3 cells, used as receiving cells, were first seeded in a 12-well plate at 1 × 10^6^ cells per well to form a single-layer of cells. Next, BHK-21 cells seeded at 4 × 10^5^ cells per well were transfected with the plasmids expressing the EGFP gene and SARS-CoV-2 spike gene of the original Wuhan strain at a ratio of 1:5 for 24 h. The cells were harvested using the Cell Dissociation Buffer (Gibco, Thermofisher, #13151014) and resuspended in serum-free DMEM (Gibco). The BHK-21 cells expressing both the EGFP and spike genes were used as donor cells; they were co-cultured on a single-layer of Calu-3 cells for cell–cell contact in the presence or absence of 500 μg/ml of honeysuckle-EtOH or honeysuckle-H_2_O, 1,000 μg/ml of APS or APS-L, and 50 μg/ml of honeysuckle-EtOH combined with 1,000 μg/ml of either APS or APS-L, and incubated at 4°C for 1 h. After 1 h, cells were washed by PBS to remove the unbound cells and replaced with the growth medium. Initial images of EGFP-positive cells, representing for binding efficiency, were acquired at five random fields using an inverted fluorescence microscope (Olympus IX70). Next, these cells were treated with the corresponding treatments and then incubated at 37°C for an additional 4 h before five fields of EGFP-positive cells were randomly imaged as previous, signifying fusion efficiency. The binding efficiency of the EGFP-positive BHK-21 cells with Calu-3 cells in the control and TCM treated groups was quantified by counting the initial number of EGFP-positive BHK-21 cells attached to Calu-3 cells. The number of EGFP cells in the control group was defined as having a binding efficiency of 100%. Thus, the effect of the TCM on the binding efficiency was determined by the percentage of binding efficiency normalized to the control. The formation of syncytial cells was calculated by quantifying the expanding area of EGFP-positive cells in the images using ImageJ. The fold-change in the EGFP-positive area in the control group from initial to 4 h was considered as a fusion efficiency of 100%. The effect of TCM on syncytia formation was calculated according to the following equation:

$$Normalized\;percentage\;\left(\%\right)=\frac{the\;fold\;change\;of\;EGFP\;area\;in\;TCM-treated\;group}{the\;fold\;change\;of\;EGFP\;area\;in\;control\;group}\times 100$$

### Enzyme-Linked Immunosorbent Assay

We performed an additional experiment using enzyme-linked immunosorbent assay (ELISA) to evaluate the honeysuckle and Huangqi’s efficacy on interfering with the binding of trimeric SARS-COV-2 Spike protein wild type (Whuan strain) or variants (α, β, γ, and δ) to biotinylated human ACE2 recombinant protein. Firstly, each well of a 96-well plate was coated with 100 μL of spike protein (500 ng/ml; cat. GTX135972-pro, GeneTex, Taipei, Taiwan) diluted in coating buffer, consisting of sodium carbonate (15 mM), sodium hydrogen carbonate (35 mM), pH 9.6, at 4°C overnight. The coated plate was then washed three times with washing buffer, consisting of PBS with 0.05% (v/v) Tween‐20 (pH 7.4) and subsequently blocked with 250 μL of blocking buffer consisting of 0.5% (w/v) bovine serum albumin (BSA) for 1.5 h at 37°C. The plate was then washed three times, then 100 μL of tested drug or inhibitor (10 μg/ml; cat. GTX635791, GeneTex, Taipei, Taiwan) in dilution buffer was added to the plate and incubated for 1 h at 37°C. Next, 100 μL of biotinylated human ACE2 protein (10 ng/ml; cat. AC2-H82E6-25ug; ACRO Biosystems, OX, UK) was added to each well and incubated for another 1 h at 37°C. The plate was then washed three times with wash buffer before 100 μL of Streptavidin-HRP conjugate (100 ng/ml; cat. GTX30949, GeneTex, Taipei, Taiwan) in dilution buffer was added and incubated for 1 h at 37°C. Afterward, the plate was washed then incubated with 200 μL of TMB substrate per well for 20 min at 37°C under light protection. Next, 50 μL of stop solution was added to terminate the reaction, and the absorbance was detected at 450 nm using a microplate reader (Cytation 5, BioTek, Vermont, United States).

### Statistical Analysis

For CPDB, predefined confidence for each set (pathway) was calculated by a series of steps. The first step was to use a hypergeometric distribution to calculate the discrete probability of the user’s inputted gene that appeared in known pathway genes recorded in the selected databases. The second step was to calculate the *p*-value of discrete probability and correct the value by false discovery rate (FDR) and q-value. Finally, one needs to quantify the fraction among possible interactions between the neighborhood-based pathways to define the connectivity index and subsequently use the index values as edges to generate visualized networks. For IPA, the overlapped rate between inputted genes and known pathway genes was calculated by Fisher’s exact test. The significant *p*‐value was set as < 0.01. Statistics significance of experimental results was calculated by Student’s t-test. *:*p* < 0.05; **, *p* < 0.01; ***, *p* < 0.001. Similar for ^#^ and ^$^.

## Results

### Big Data Analysis Predicts Honeysuckle and Huangqi as TCM Candidates for Prevention and Treatment COVID-19

We comprehensively reviewed the current medications and investigational drugs for COVID-19 using UpToDate and DynaMed databases. We identified 38 drugs or compounds as follows: glucocorticoids, including dexamethasone, hydrocortisone, and methylprednisolone; anti-viral agents, such as remdesivir, favipiravir, and ritonavir; immunomodulators including hydroxychloroquine and chloroquine; JAK inhibitors such as baricitinib; antiparasitic drugs including ivermectin; and other compounds under investigation, such as azithromycin, fluvoxamine, and famotidine (Table 1). Of these 38 drugs, 25 drugs could retrieve their corresponding target genes (Figure 1A) from CLUE database. A total of 30 target genes was obtained (Figure 1B; Table 1). We also sorted the inflammatory signaling pathways in COVID-19-related ARDS, including those of IL-6/JAK/STAT, interferon, NF-κB, TLRs, Bruton’s tyrosine kinase, and renin–angiotensin system (Zhang and Guo, 2020; Choudhary et al., 2021), and 49 target genes were included for further analysis (Figure 1C). The union of 30 and 49 target genes were used to searching for their corresponding TCM drugs *via* SymMap (Supplementary Tables S1, S2; filtered by *p* < 0.05). Each target gene may connect to different number of TCM drugs. For example, *TNF*, a highly frequent gene appearing in SymMap, connected up to 441 TCM drugs, whereas *TLR3* only linked to 1 TCM. We then selected 10 highly frequent target genes (each connection with > 200 TCMs for each gene) and intersected their TCM sets to obtain 66 TCM candidates, which might potentially inhibit ARDS and COVID-19-related inflammatory response (Figures 1D,E; Table 2). Therefore, every single TCM listed in Table 2 links to 10 highly frequent target genes, including *TNF*, *CYP1A2*, *CYP3A4*, *NOS2*, *IL6*, *IL1B*, *STAT1*, *NFKBIA*, *CXCL10*, and *IFNG*. These 66 TCM candidates included a broad spectrum of common therapeutic classes, including antipyretics, antitussives, antiasthmatics, and Qi-reinforcing drugs. However, most of the predicted TCM drugs have not been thoroughly tested their inhibitory activities against SARS-CoV-2 (Lee et al., 2021; Jia et al., 2021). To prioritize potential candidates from these 66 TCM candidates, the following three criteria were considered: Firstly, we hypothesized that *in silico* identified potential anti-SARS-CoV-2 TCMs covering a wide range of pharmacologic functions with minimal side effects (Table 2) could be integrated into our clinical practice. Secondly, two reports utilized statistical calculation of the frequently used TCMs for SARS-CoV-2 infection in China (Luo et al., 2020; Xu et al., 2020). They identified 19 frequently used TCM in COVID-19 treatment. Among them, *Lonicera Japonica* (honeysuckle) and *Astragalus membranaceus* (Huangqi) were overlapped with our 66 TCM list. Despite some TCMs are reported to contain nephrotoxins and mutagens (Ng et al., 2017), the toxicology of most of TCMs remains to be determined (Zeng and Jiang, 2010). Instead, Honeysuckle and Huangqi showed safety without distinct toxicity or side effects in various studies (Shang et al., 2011; Fu et al., 2014). Finally, our TCM combination includes the ingredients of heat-toxin clearing (honeysuckle) and qi-tonifying (Huangqi) comparing to other clinical trial medicines, which mainly consist of heat-toxin clearing agents and ignore the value of TCM in providing a supportive role in the treatment of COVID-19. Hence, we selected two low toxicities TCM drugs, honeysuckle and Huangqi, to further evaluate their anti-viral activities *in silico* and *in vitro*.

**TABLE 1:** Current COVID-19 drugs and their target genes.

Current medication (25)	Target genes
Dexamethasone	*ANXA1*, *CYP3A4*, *CYP3A5*, *NOS2*, *NR0B1*, *NR3C1*, *NR3C2*
Betamethasone	*NR3C1*
Prednisone	*HSD11B1*, *NR3C1*
Methylprednisolone	*NR3C1*
Hydrocortisone	*ANXA1*, *NOS2*, *NR3C1*, *NR3C2*
Clobetasol	*NR3C1*, *PLA2G1B*
Diflorasone	*NR3C1*, *PLA2G1B*
Fluocinonide	*NR3C1*, *SERPINA6*, *SMO*
Halobetasol	*NR3C1*, *PLA2G1B*
Amcinonide	*ANXA1*, *NR3C1*
Desoximetasone	*NR3C1*, *PLA2G1B*
Halcinonide	*NR3C1*
Triamcinolone	*CYP3A5*, *CYP3A7*, *NR3C1*, *SERPINA6*
Clocortolone	*NR3C1*, *PLA2G1B*
Fluocinolone	*NR3C1*, *SERPINA6*
Flurandrenolide	N/A
Fluticasone	N/A
Mometasone	*NR3C1*
Prednicarbate	*NR3C1*, *PLA2G1B*
Alclometasone	*NR3C1*, *SERPINA6*
Remdesivir	N/A
Baricitinib	N/A
Tocilizumab	N/A
Sarilumab	N/A
Siltuximab	N/A
**Investigational treatment (13)**	**Target Genes**
Hydroxychloroquine	*TLR7*, *TLR9*
Chloroquine	*CYP2C8*, *GSTA2*, *MRGPRX1*, *TLR9*, *TNF*
Favipiravir	N/A
Anakinra	N/A
Azithromycin	*MLNR*
Lopinavir	N/A
Ritonavir	*CYP1A2*, *CYP2B6*, *CYP2C19*, *CYP3A4*, *CYP3A5*, *CYP3A7*
Ivermectin	*CHRNA7*, *GABRB3*, *GLRA3*, *P2RX7*
Sofosbuvir	N/A
Daclatasvir	N/A
Fluvoxamine	*CYP2C19*, *SLC6A4*
Famotidine	*HRH2*
Zinc	N/A

*N/A not available in CLUE database.

**TABLE 2:** The scientific name, Chinese name, and therapeutic category of the TCM candidates predicted to have anti-SARS-CoV-2 effects.

Scientific name	Chinese name	Pinyin name	Latin name	Category
*Lonicera japonica*	金銀花	Jinyinhua	Lonicerae Japonicae Flos	Antipyretic Detoxicate Drugs
*Astragalus membranaceus*	黃耆	Huangqi	Astragali Radix	Qi Reinforcing Drugs
*Forsythia suspensa*	連翹	Lianqiao	Forsythiae Fructus	Antipyretic Detoxicate Drugs
*Ephedra sinica*	麻黃	Mahuang	Ephedrae Herba	Pungent-Warm Exterior-Releasing Medicinal
*Inula japonica*	金沸草	Jinfeicao	Inulae Herba	Phlegm resolving Medicine
*Chrysanthemum morifolium*	菊花	Juhua	Chrysanthemi Flos	Pungent Cool Diaphoretics
*Bupleurum chinensis*	柴胡	Chaihu	Bupleuri Radix	Pungent Cool Diaphoretics
*Aster tataricus*	紫菀	Ziwan	Asteris Radix Et Rhizoma	Antitussive Antiasthmetics
*Salvia miltiorrhiza*	丹參	Danshen	Salviae Miltiorrhizae Radix Et Rhizoma	Blood Activating Stasis Removing Drugs
*Patrinia scabiosaefolia*	敗醬草	Baijiangcao	Patriniae Herba	Antipyretic Detoxicate Drugs
*Aloysia Citriodora*	馬鞭草	Mabiancao	Verbenae Herba	Blood Activating Stasis Removing Drugs
*Portulaca oleracea*	馬齒莧	Machixian	Portulacae Herba	Antipyretic Detoxicate Drugs
*Centipeda minima*	鵝不食草	Ebushicao	Centipedae Herba	Pungent-Warm Exterior-Releasing Medicinal
*Morus alba*	桑白皮	Sangbaipi	Mori Cortex	Antitussive Antiasthmetics
*Agrimonia pilosa*	仙鶴草	Xianhecao	Agrimoniae Herba	Astringent Hemostatic Medicinal
*Phyllanthus emblica*	余甘子	Yuganzi	Phyllanthi Fructus	Antipyretic Detoxicate Drugs
*Illicium verum*	八角茴香	Bajiaohuixiang	Anisi Stellati Fructus	Warming Interior Drugs
*Speranskia tuberculata*	鳳仙透骨草	Fengxiantougucao	Speranskiae Tuberculatae Herba	Wind-Dampness Dispelling And Cold Dispersing Medicinal
*Lobelia chinensis*	半邊蓮	Banbianlian	Herba Lobeliae Chinensis	Antipyretic Detoxicate Drugs
*Ilex chinensis*	四季青	Sijiqing	Ilicis Chinensis Folium	Antipyretic Detoxicate Drugs
*Euphorbia humifusa*	地錦草	Dijincao	Herba Euphorbiae Humifusae	Antipyretic Detoxicate Drugs
*Prunella vulgaris*	夏枯草	Xiakucao	Spica Prunellae	Fire Purging Drugs
*Hydnocarpus anthelmintica*	大風子	Dafengzi	Hydnocarpus Anthelmintica Semen	Medicinal For Detoxification, Parasiticide, Drying Dampness And Relieving Itching
*Saussurea involucrata*	天山雪蓮	Tianshanxuelian	Saussureae Involucratae Herba	Yang Reinforcing Drugs
*Potentilla chinensis*	委陵菜	Weilingcai	Potentiliae Chinensis Herba	Antipyretic Detoxicate Drugs
*Sophora tonkinensis*	山豆根	Shandougen	Sophorae Tonkinensis Radix Et Rhizoma	Antipyretic Detoxicate Drugs
*Microcos paniculata*	布渣葉	Buzhaye	Microctis Folium	External Medicinal (Draw Out Toxin, Resolve Putridity)
*Choerospondias axillaris*	廣棗	Guangzao	Choerospondiatis Fructus	Blood Activating Stasis Removing Drugs
*Rosa chinensis*	月季花	Yuejihua	Rosae Chinensis Flos	Blood Activating Stasis Removing Drugs
*Oroxylum indicum*	木蝴蝶	Muhudie	Oroxyli Semen	Antipyretic Detoxicate Drugs
*Equisetum hiemale*	木賊	Muzei	Equiseti Hiemalis Herba	Pungent Cool Diaphoretics
*Eucommia ulmoides*	杜仲葉	Duzhongye	Eucommiae Folium	External Medicinal (Draw Out Toxin, Resolve Putridity)
*Hovenia dulcis*	枳椇子	Zhijuzi	Hovenia Dulcis Fructus	Diuretic Dampness Excreting Drugs
*Ilex cornuta*	枸骨葉	Gouguye	Ilicis Cornutae Folium	Asthenic Heat Dispelling Drugs
*Diospyros kaki*	柿蒂	Shidi	Kaki Calyx	Qi Regulating Drugs
*Gardenia jasminoides*	梔子	Zhizi	Gardeniae Fructus	Fire Purging Drugs
*Morus alba*	桑椹	Sangshen	Mori Fructus	Yin-Tonifying Medicinal
*Hippophae rhamnoides*	沙棘	Shaji	Hippophae Fructus	Phlegm resolving Medicine
*Euphorbia helioscopia*	澤漆	Zeqi	Euphorbia Helioscopia Herba	Diuretic Dampness Excreting Drugs
*Gleditsia sinensis*	皂角刺	Zhaojiaoci	Gleditsiae Spina	Phlegm resolving Medicine
*Leonurus heterophyllus*	益母草	Yimucao	Leonuri Herba	Blood Activating Stasis Removing Drugs
*Punica granatum*	石榴皮	Shiliupi	Granati Pericarpium	Astringent Medicinal
*Carthamus tinctorius*	紅花	Honghua	Carthami Flos	Blood Activating Stasis Removing Drugs
*Potentilla discolor*	翻白草	Fanbaicao	Potentillae Discoloris Herba	Antipyretic Detoxicate Drugs
*Geranium wilfordii*	老鸛草	Laoguancao	Geranii Herba	Wind-Dampness Dispelling And Cold Dispersing Medicinal
*Daphne genkwa*	芫花	Yuanhua	Genkwa Flos	Drastic Purgatives
*Litsea cubeba*	蓽澄茄	Bichengqie	Litseae Fructus	Warming Interior Drugs
*Nelumbo nucifera*	荷葉	Heye	Nelumbinis Folium	Antipyretic Detoxicate Drugs
*Pueraria lobata*	葛花	Gehua	Puerariae Lobatae Flos	Pungent Cool Diaphoretics
*Typha angustifolia*	蒲黃	Puhuang	Typhae Pollen	Stasis-Resolving Hemostatic Medicinal
*Prinsepia uniflora*	蕤仁	Ruiren	Prinsepiae Nux	Pungent Cool Diaphoretics
*Hypericum perforatum*	貫葉金絲桃	Guanyejinsitao	Hyperici Perforati Herba	Astringent Hemostatic Medicinal
*Glechoma hederacea*	連錢草	Lianqiancao	Glechomae Herba	Diuretic Dampness Excreting Drugs
*Prunus japonica*	郁李仁	Yuliren	Pruni Semen	Laxatives
*Rosa laevigata*	金櫻子	Jinyingzi	Rosae Laevigatae Fructus	Astringent Medicinal
*Lysimachia christinae*	金錢草	Jinqiancao	Lysimachiae Herba	Diuretic Dampness Excreting Drugs
*Uncaria rhynchophylla*	鉤藤	Gouteng	Uncariae Ramulus Cumuncis	Liver-Pacifying Wind-Extinguishing Medicinal
*Clematdis intricata*	鐵線透骨草	Tiexiantougucao	Clematdis Intricata Herba	Wind-Dampness Dispelling And Cold Dispersing Medicinal
*Ginkgo biloba*	銀杏葉	Yinxingye	Ginkgo Folium	Antitussive Antiasthmetics
*Artemisia annua*	青蒿	Qinghao	Artemisiae Annuae Herba	Asthenic Heat Dispelling Drugs
*Elsholtzia ciliata*	香薷	Xiangru	Moslae Herba	Pungent-Warm Exterior-Releasing Medicinal
*Cyperus rotundus*	香附	Xiangfu	Cyperi Rhizoma	Qi Regulating Drugs
*Alpinia offinarum*	高良薑	Gaoliangjiang	Alpiniae Officinarum Rhizoma	Warming Interior Drugs
*Paederia foetida*	雞屎藤	Jishiteng	Paederia Foetida Rhizoma	Digestants
*Ephedra sinica*	麻黃根	Mahuanggen	Ephedrae Radix Et Rhizoma	Astringent Medicinal
*Gnaphalium affine*	鼠麴草	Shuqucao	Gnaphalium Affine Herba	Phlegm resolving Medicine

Identification of potential TCM candidates for COVID-19 treatment. The target genes indicate the biological function affected by the drug treatment and viral mechanisms. Therefore, small-molecule reagents, viral mechanisms, and the TCM candidates can be linked through the common shared target genes. The flow chart describes the steps in the bioinformatics analysis.

### The Potential Mechanism of Honeysuckle, Huangqi, and miRNA (Let-7a and miR-148b) in Suppressing SARS-Co-V2 Infection

We investigated honeysuckle and Huangqi’s biological function by predicting their potential mechanisms with multiple target-pathway interaction databases. First, we screened for highly potential targets of honeysuckle and Huangqi in the SymMap database (FDR-BH < 0.01) and we acquired 66 and 64 potential target genes of honeysuckle and Huangqi, respectively. These targets were further analyzed with two enrichment analysis methods, CPDB and IPA, to reveal the possible mechanisms.

**FIGURE 2:**
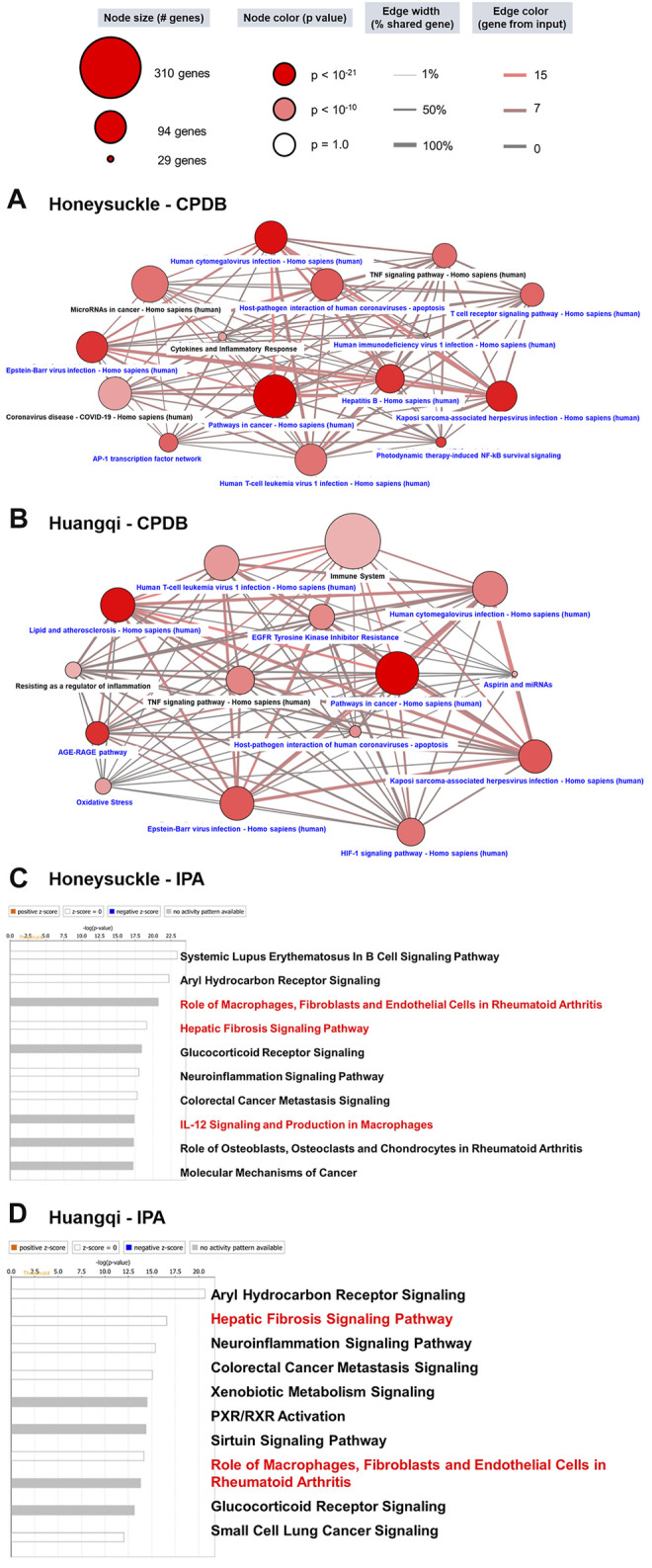
The potential mechanism of Honeysuckle (*Lonicera japonica*) and Huangqi (*Astragalus membranaceus*) *via* the bioinformatics workflow. **(A)** According to the results of the FDR-BH statistical analysis, we selected 66 significant honeysuckle targets to perform enrichment analysis *via* CPDB. To begin, 15 pathways were selected from the top 150 pathways (ranked by *p*-value < 0.001) using the following keywords: virus, viral, infection, microRNA (miRNA), immune, inflammation, TNF, interleukin (IL), interferon (IFN), cytokine, etc. Those pathways highlighted in black were focused upon to discuss the possible therapeutic potential of TCMs. The 15 selected pathways in this study were illustrated in the context of a network. The dot color denotes statistical significance where the darker color demonstrates a higher significance level. The dot size indicates the number of genes in the pathway, while the edge between two dots showed the relationship. **(B)** The 64 target genes of Huangqi were analyzed though the same process in CPDB. The bar chart shows the results of the IPA analysis of honeysuckle **(C)** and Huangqi **(D)**. Since the target genes in this study were retrieved from a database or PubMed, there were no up- or down-regulated expression data. Thus, we predicted the potential pathway without up- or down-regulation. A white bar color (no activation change) or gray (unknown activation) was the degree of correlation with the input gene set. The pathways of interest in this study were highlighted red.

According to the CPDB network diagram, honeysuckle could interfere with various viruses, such as hepatitis B, herpes, Epstein–Barr, and hepatitis C viruses. Interestingly, this herb mediated the cytokine and inflammation response and NF-κB signaling pathways, both of which were connected to cytokine storm symptoms and were regarded as a critical syndrome in SARS-CoV-2 infection (Choudhary et al., 2021) (Figure 2A). Similarly, Huangqi network from CPDB also included viruses’ pathological pathways and inflammation signaling (Figure 2B). On the other hand, IPA reported that honeysuckle was highly related to ARDS pathological mechanisms, including immune system *via* B cell and macrophages, IL-6/8/12, and fibrosis (Choudhary et al., 2021) (Figure 2C). Meanwhile, the potential pathways of Huangqi were analyzed *via* the same bioinformatics pipeline and one of the pathways was related to fibrosis and the macrophage activation (Figure 2D). Thus, the cross-databases validation not only strengthened the bioinformatics prediction between CPDB and IPA but also prioritized honeysuckle as the TCM candidate for preventing and treating COVID-19. The statistical values of each pathway were listed in Supplementary Table S3 (CPDB) and 4 (IPA). Moreover, *IL-6* and *TNF* were the important factors in the cytokine storm and were upregulated in the expression profile of Calu-3 with SARS-CoV-2 infection (Supplementary Figure S3). These data suggested that SARS-CoV-2 pathological processes and honeysuckle-associated signaling were closely intertwined; therefore, honeysuckle could be a promising herbal treatment for COVID-19.

**FIGURE 3:**
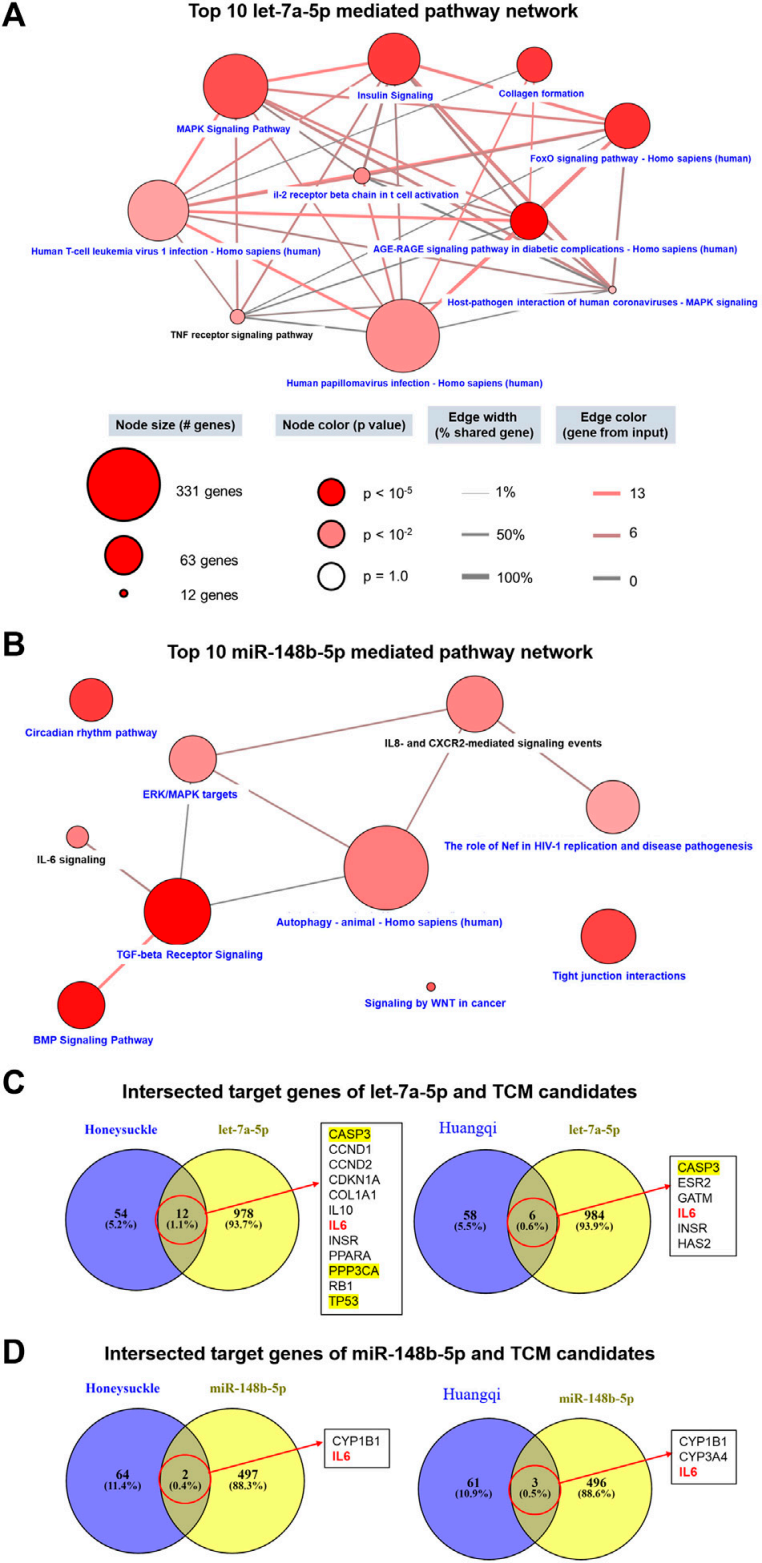
Prediction of the functions of *let-7a* and *miR-148b* and their connections with the TCM candidates using multiple databases. miRNA-related targets were obtained from miRDB, and each list of genes (990 target genes for *let-7a-5p* and 499 target genes for *miR-148b-5p*) were used to query CPDB for signaling pathways. **(A,B)** show the network of 10 signaling pathways related to this study and mediated by *let-7a-5p* and *miR-148b-5p*, respectively. Pathways in black are highly related to this study. **(C,D)** display the intersecting target genes of miRNA and TCM candidates. Of particular interest, the MAPK pathway (yellow) and the IL6 pathway (red) are associated with cytokine storms in COVID-19 patients.

miRNA is a critical modulator in the pathogenesis of virus infection; thus, we also considered the miRNA-mediated mechanisms as another COVID-19 therapeutic target. We investigated the mechanisms associated-miRNA by using the miRDB website to identify miRNA-targeted genes. A total of 990 target genes of *let-7a-5p*, 499 target genes of *miR-148b-5p*, and 488 target genes of miR-146a-5p were input to CPDB for enrichment analysis. The network of the top 10 pathways mediated by *let-7a-5p* had a connection to inflammation, such as MAPK, PI3K-Akt, and FoxO signaling, and fibrosis, such as collagen and AGR-RAGE signaling (Figure 3A). Among them, the TNF receptor-signaling pathway was identified in our study. Moreover, these top 10 *let-7a-5p* mediated pathways shared a certain degree of correlation between each other. In contrast, the connection between the top 10 *miR-148b-5p* mediated pathways was loose. As there were 7 out of 10 pathways was related to infection or lymphocyte immunity, such as IL-6, IL-8, CXCR2, TGF-β, autophagy, and HIV-1 replication, whereas BMP singling was associated with extracellular interactions (Figure 3B).

We delineated the relationship between miRNAs and the TCM candidates by intersecting their respective target genes. Honeysuckle and Huangqi shared 12 and 6 common target genes with *let-7a-5p* (Figure 3C), respectively. For example, *CASP3*, *TP53*, and *PPP3CA* were members of the MAPK pathway, while *IL6* and *IL10* were members of the cytokine family. As for the association between *miR-148b-5p* and the TCM candidates, *IL6* was one of the common intersected target gene between them (Figure 3D).

Both honeysuckle and Huangqi were suggested by our analysis to have potential association with *miR-146a-5p* (Supplementary Table S5). As for the top 10 miR-146a-5p mediated pathway networks, most of them were related to cell proliferation and survival (RAC1, Wnt, NOTCH, RNA polymerase II, and transcription pathways), whereas TCR signaling and Herpes simplex virus 1 infection may relate to an immune reaction or inflammation (Supplementary Figure S4A). Among them, the NOTCH pathway was linked to COVID-19 pathophysiology and cardiovascular complications (Breikaa and Lilly, 2021). The intersected target genes of TCM candidates and *miR-146a-5p* were *PTGS2* and *PSMD3* (Supplementary Figure S4B). PTGS2, mediating the peroxidase in the biosynthesis pathway, has a crucial role in the inflammatory response. On the other hand, PSMD3, a component of the 26S proteasome, cleaves peptides on the non-lysosomal pathway. Therefore, the involvement of these two genes implied that our TCM candidates might target to the processes associated with the virus’ life cycle progression.

### The EtOH and H_2_O Extractions of Honeysuckle and Huangqi Elevated Let-7a, miR-148b, and miR-146a Expression

Since elevating the expression of *let-7a*, *miR-148b*, and *miR-146a* was likely beneficial for treating COVID-19 (Sabbatinelli et al., 2021; Xie et al., 2021), we studied the effects of the TCM candidates on the expression of these miRNAs. First, we determined the highest safe dosage of the TCM candidates by treating BEAS2B cells with different concentrations of the candidates for 24 h in 96-well plates and evaluated cell viability using the SRB assay. The cells treated with honeysuckle-H_2_O at 500 μg/ml and two different Huangqi extracts (APS and APS-L) at 1 mg/ml each maintained a viability rate of more than 80%. However, the cells treated with honeysuckle-EtOH 500 μg/ml had the lowest survival rate at about 70%, still an acceptable rate (Supplementary Figures S5A,B).

**FIGURE 4:**
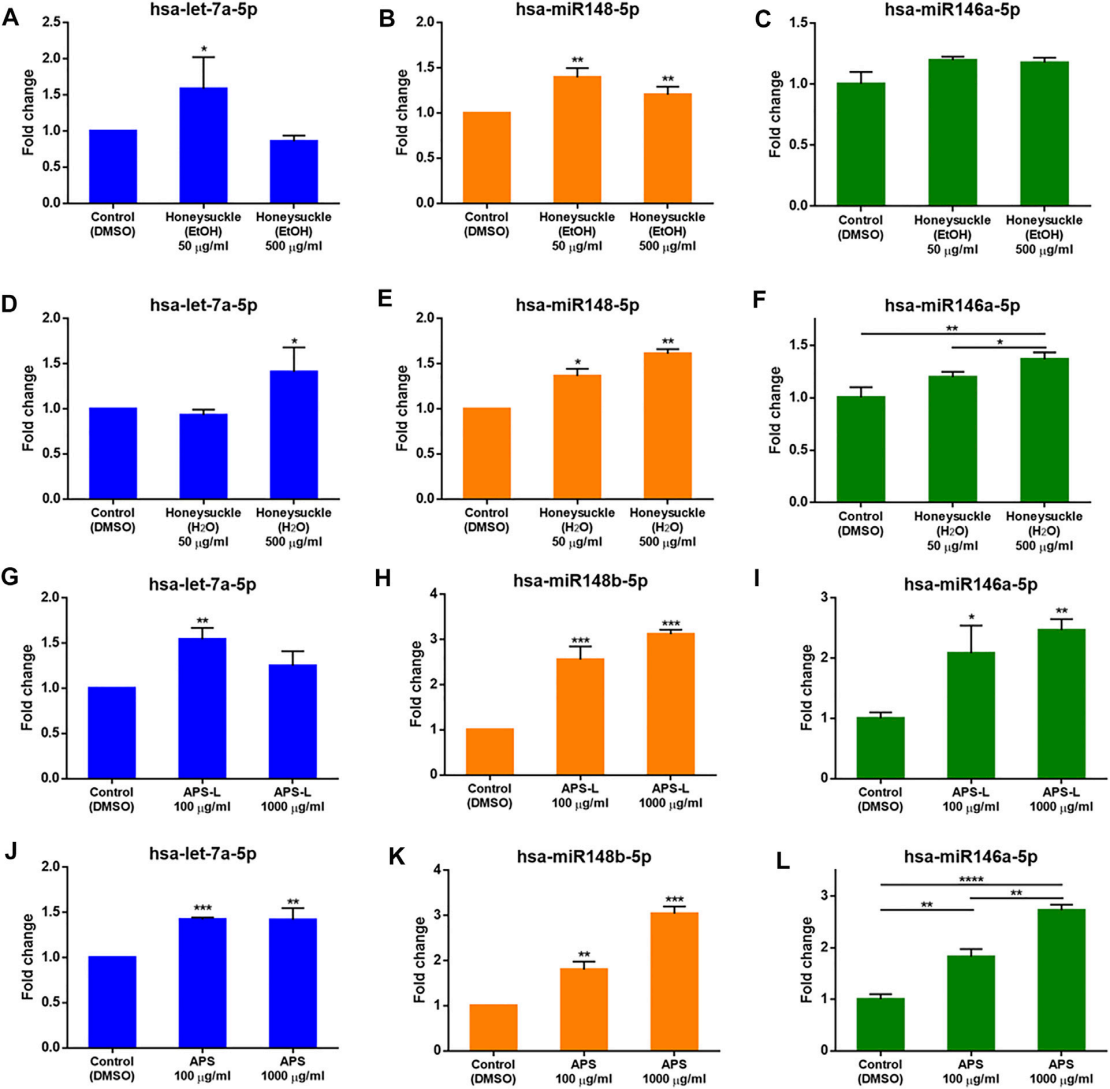
Induction of *let-7a*, *miR-148b*, and *miR-146a* levels by the TCM candidates. *let-7a*, *miR-148b*, and *miR-146a* levels were measured by qRT-PCR normalized to the internal control U54 after a 24-h treatment with honeysuckle-EtOH **(A-C)**, honeysuckle-H2O **(D-F)**, APS-L **(G-I)**, and APS **(J-L)**. Data are expressed as mean ± SD from three repeated results and analyzed using Student’s t-test. ^*^, significantly different from the corresponding control, at p < 0.05; ^**^, p < 0.01; ^***^, p < 0.001.

At 50 μg/ml, honeysuckle-EtOH could induce both *let-7a* and *miR-148b* expression by 1.6- and 1.4-fold (Figures 4A,B), respectively, while at its highest dose of 500 μg/ml, it could only increase *miR-148b* expression by 1.2-fold (Figure 4E). On the other hand, *miR-146a* was only slightly elevated by these treatments (Figure 4C). Meanwhile, *let-7a* expression was activated only by a high dose of honeysuckle-H_2_O by 1.4-fold (Figure 4D), whereas *miR-148b* and *miR-146a* were gradually upregulated by 50 and 500 μg/ml honeysuckle-H_2_O by 1.4- and 1.6-fold and 1.2- and 1.4-fold, respectively (Figures 4E,F). In contrast, all the treatments of Huangqi extracts could enhance the manifesting level of targeted miRNAs. Both APS and APS-L could increase the level of *let-7a* by 1.2- to 1.5-fold, respectively (Figures 4G,J), while they were more effective in upregulating *miR-148b* and *miR-146a* by approximately 2- to 3-fold, respectively (Figures 4H,I,K,L). These results suggested that both honeysuckle and Huangqi could increase the expression of *let-7a*, *miR-148b*, and *miR-146a.* The miRNA profiles of mice and human volunteers after ingestion of honeysuckle were investigated, and *let-7a* and *miR-148b* were significantly overexpressed (Lee et al., 2017; Lee et al., 2021). BEAS2B cells were used to validate our previous *in vivo* investigation. Despite the values of miRNA induction was not dramatic, they all reached statistical significance, indicating that them may have effect on biological functions.

### Validation Cytokine Storm Inhibiting Ability on Immune Cells of Candidate TCM

**FIGURE 5:**
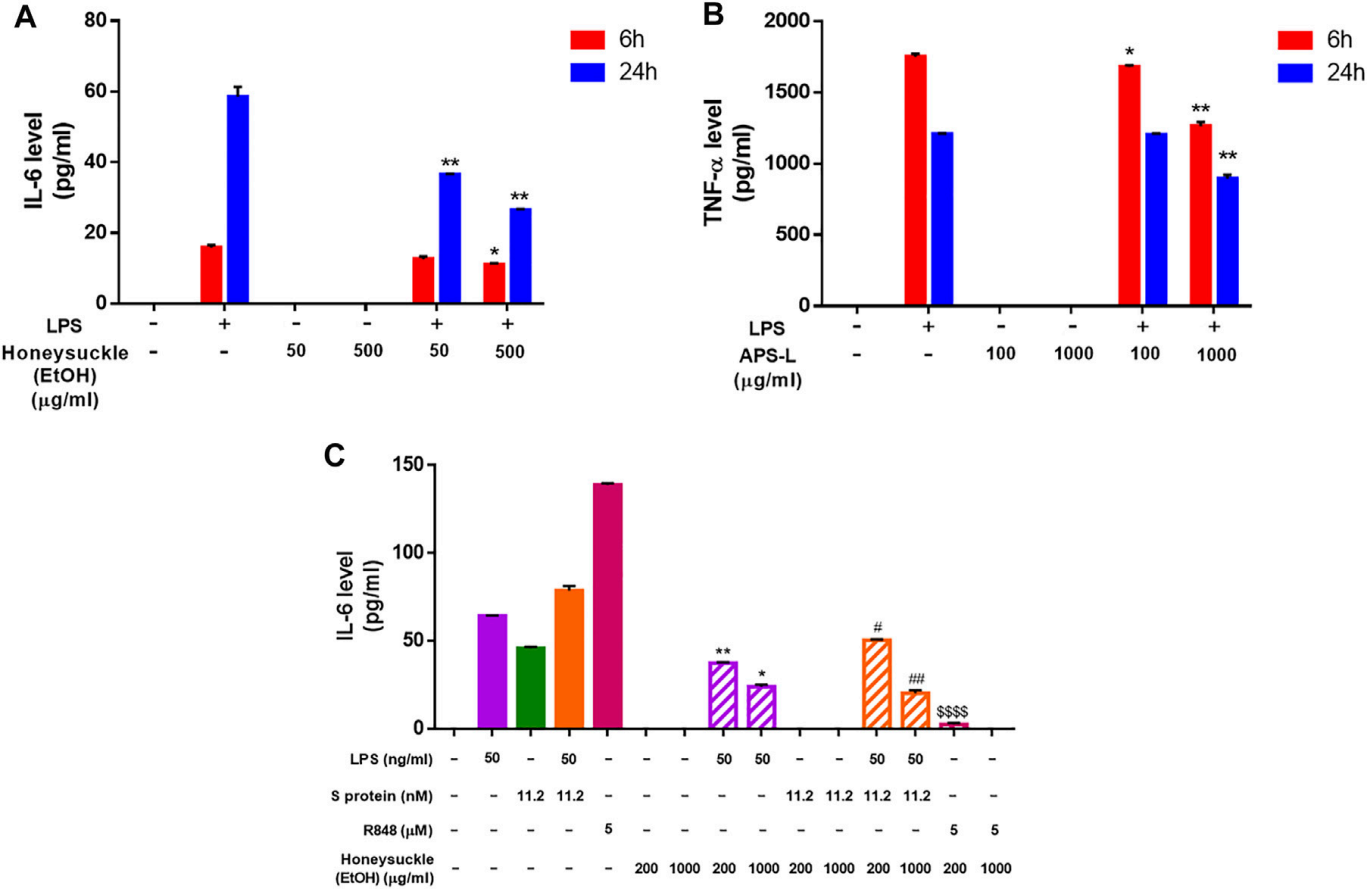
Inhibition of cytokine release level by the TCM candidates. After 6 and 24 h of honeysuckle-EtOH **(A)** or APS-L **(B)** treatment in the presence or absence of LPS, THP-1 cell medium was collected, and ELISA was performed to measure cytokine levels. Treatment with LPS alone was considered to be the control. **(C)** After 24 h of honeysuckle-EtOH treatment in the presence or absence of stimulators (LPS, Spike, LPS + Spike, and R848), THP-1 cell medium was collected, and ELISA was performed to measure IL-6 levels. Data are expressed as mean ± SD from three repeated results and analyzed using Student’s t-test. ^*^ compared to LPS stimulation alone, ^#^ compared to combined stimulation of LPS and Spike, and ^$^ compared to R848 stimulation alone. ^*^, significantly different from the corresponding control, at p < 0.05; ^**^, p < 0.01. Similar for ^#^ and ^$^.

After 6 or 24 h of honeysuckle or Huangqi treatment, the cell medium was collected to quantify the secretion level of IL-6 and TNF-α, two of the most abundantly detected cytokines in COVID-19 patients’ plasma (Choudhary et al., 2021). The treatment with stimulator LPS alone was used as the control. Because LPS is a potent immune stimulus that causes cytokine storm, LPS stimulation was used as a model to investigate the capability of honeysuckle and Huangqi treatments in the inhibition of cytokine productions. The honeysuckle-EtOH treatment demonstrated inhibition of IL-6 secretion. Both tested doses of honeysuckle-EtOH suppressed the release of IL-6 in the presence of LPS at both time points. Still, honeysuckle-EtOH 500 μg/ml displayed more prominent effect on suppressing IL-6 than lower dose did (Figure 5A). These data indicated that honeysuckle-EtOH could downregulate LPS-induced IL-6 secretion. Remarkably, APS-L could inhibit TNF-α, as predicted by *in silico* analysis (Supplementary Table S3C). In the inflammatory environment, APS-L at 100 and 1,000 μg/ml could suppress TNF-α in a time- and dose-dependent manner. While the higher dose of APS-L could cause a noticeable inhibition of TNF-α release at both time points, the lower dose could only lower the secretion level of TNF-α slightly (Figure 5B).

On the other hand, to determine the efficacy of honeysuckle on inhibiting cytokine storm which is induced by SARS-CoV-2 infection, we used recombinant S protein and R848, a TLR7/8 agonist as stimulators. Differentiated THP-1 cells were co-treated by recombinant S protein 11.2 nM or R848 5 µM with honeysuckle 200 or 1,000 μg/ml after being pre-treated by the corresponding dose of honeysuckle for 2 h. After 24 h of co-treatment, the cell medium was collected to quantify the secretion level of IL-6. The honeysuckle-EtOH treatment demonstrated inhibition of IL-6 secretion. Both tested doses of honeysuckle-EtOH significantly suppressed the release of IL-6 in the presence of LPS or LPS plus S protein, while S protein- and R848-induced IL-6 level was completely inhibited by both doses and high dose of honeysuckle-EtOH, respectively (Figure 5C).

These data suggested that these drug candidates were able to inhibit cytokine storms by reducing the release of IL-6 or TNF-α, which were abundant in acute-phase COVID-19 patients.

### Suppression of SARS-CoV-2 M^pro^ Activity by Honeysuckle and Huangqi

The proteolytic cleavage of SARS-CoV-2 polyproteins pp1a and pp1ab by M^pro^ residing in nsp5 releases nsp5-16 and the carboxy (C) terminus of nsp4, whose functions are necessary for viral replication (V'Kovski et al., 2021). Thus, SARS-CoV-2 M^pro^ is a promising target for therapeutic intervention against COVID-19. Therefore, the peptide sequence at the cleavage site between nsp4 and nsp5 has been synthesized into a fluorogenic peptide (Abz-TSAVLQSGFRK-Dnp) with a fluorophore (Abz) and its quencher (Dnp) for measuring the protease activity of SARS-CoV-2 M^pro^.

**FIGURE 6:**
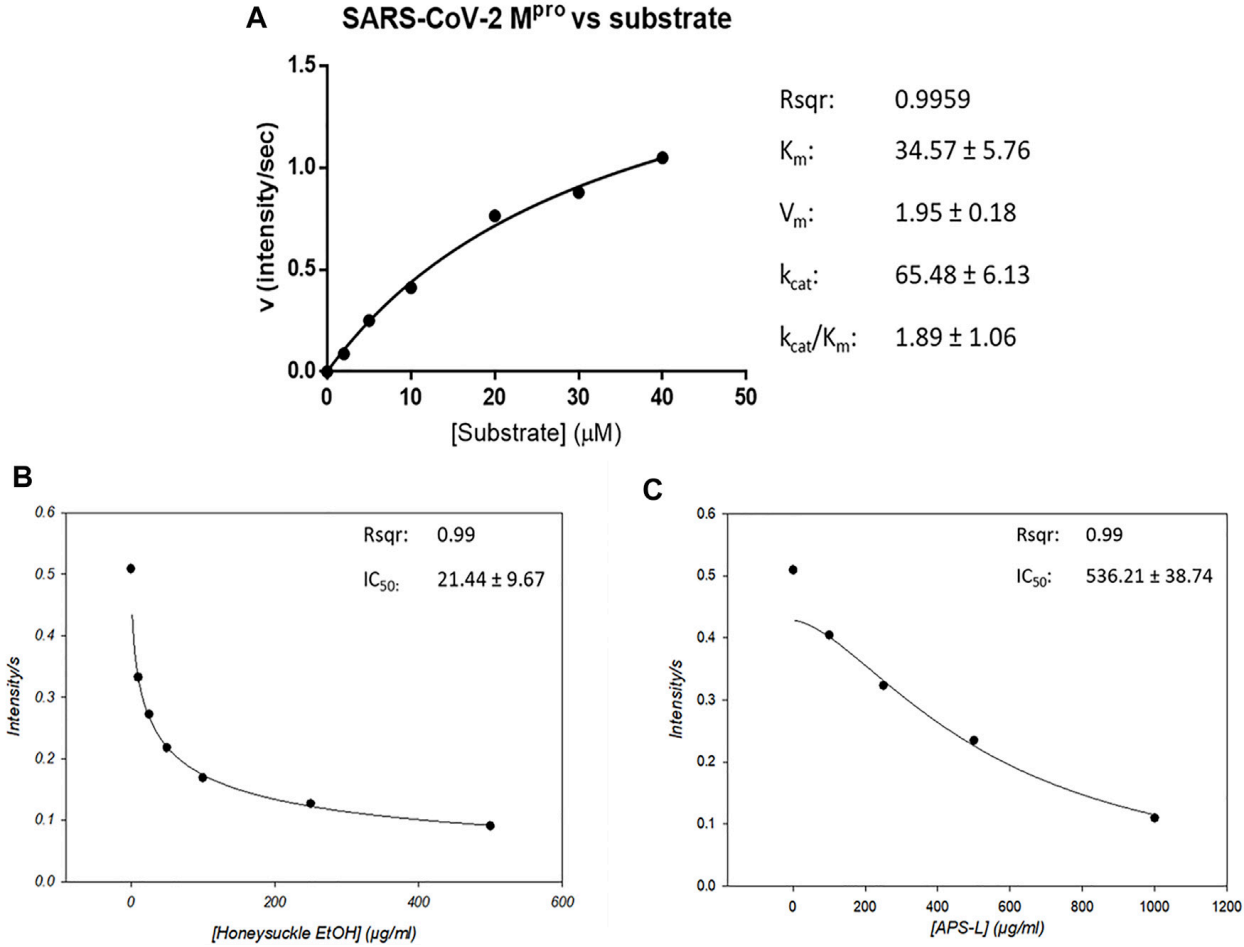
Honeysuckle and APS-L inhibit SARS-CoV2 M^pro^ activity. **(A)** Activity of SARS-CoV-2 M^pro^ in the presence of a peptide substrate, a fluorogenic probe (Abz-TSAVLQSGFRK-Dnp). The concentrations of the peptide substrate varied from 2 to 40 µM in PBS buffer, while M^pro^ concentration was fixed at 0.12 µM. The effect of honeysuckle-EtOH **(B)** and APS-L **(C)** on SARS-CoV-2 M^pro^ activity was investigated. The IC_50_ value for each reaction was calculated and displayed.

We determined the protease activity of a recombinant SARS-CoV-2 M^pro^ using this fluorogenic probe and calculated its kinetic parameters using the Michaelis–Menten equation. M^pro^’s max reaction velocity (V_m_) was 1.95 ± 0.18 intensity/sec, its Michaelis constant Km was 34.57 ± 5.76 mol/L, its turnover number K_cat_ was 65.48 ± 6.13/sec, and its K_cat_/K_m_ was 1.89 ± 1.06. The coefficient of determination (Rsqr) in this regression model was 0.9959 (Figure 6A). Then, we used this protease activity assay to examine the repression of M^pro^ activity by honeysuckle and APS. Our data showed that honeysuckle-EtOH and APS-L noticeably inhibited M^pro^ activity with the IC_50_ of 21.44 ± 9.67 μg/ml (Figure 6B) and 536.21 ± 38.74 μg/ml (Figure 6C), respectively, indicating that these two herb extracts could suppress SARS-CoV-2 M^pro^ activity.

### Suppression of the Binding of SARS-CoV-2 Spike Protein to ACE2 Receptor and the Formation of Syncytium by Honeysuckle, Huangqi, and Their Combination

Receptor-dependent syncytia formation is triggered by SARS-CoV-2 spike (S) protein on the cell membrane (Buchrieser et al., 2020; Cheng et al., 2020; Li X et al., 2020). Thus, we evaluated the anti-SARS-CoV-2 activity of honeysuckle and APS by measuring the binding efficiency between spike protein (BHK-21 cells expressing SARS-CoV-2 S protein and EGFP) and its corresponding receptor protein (Calu-3 cells expressing endogenous hACE2 receptor). The binding of BHK-21 cells to Calu-3 cells indicated the binding of the SARS-CoV-2 S protein with the ACE2 receptor. Moreover, the formation of syncytium resulting from the membrane fusion between BHK-21 and Calu-3 cells was measured.

**FIGURE 7:**
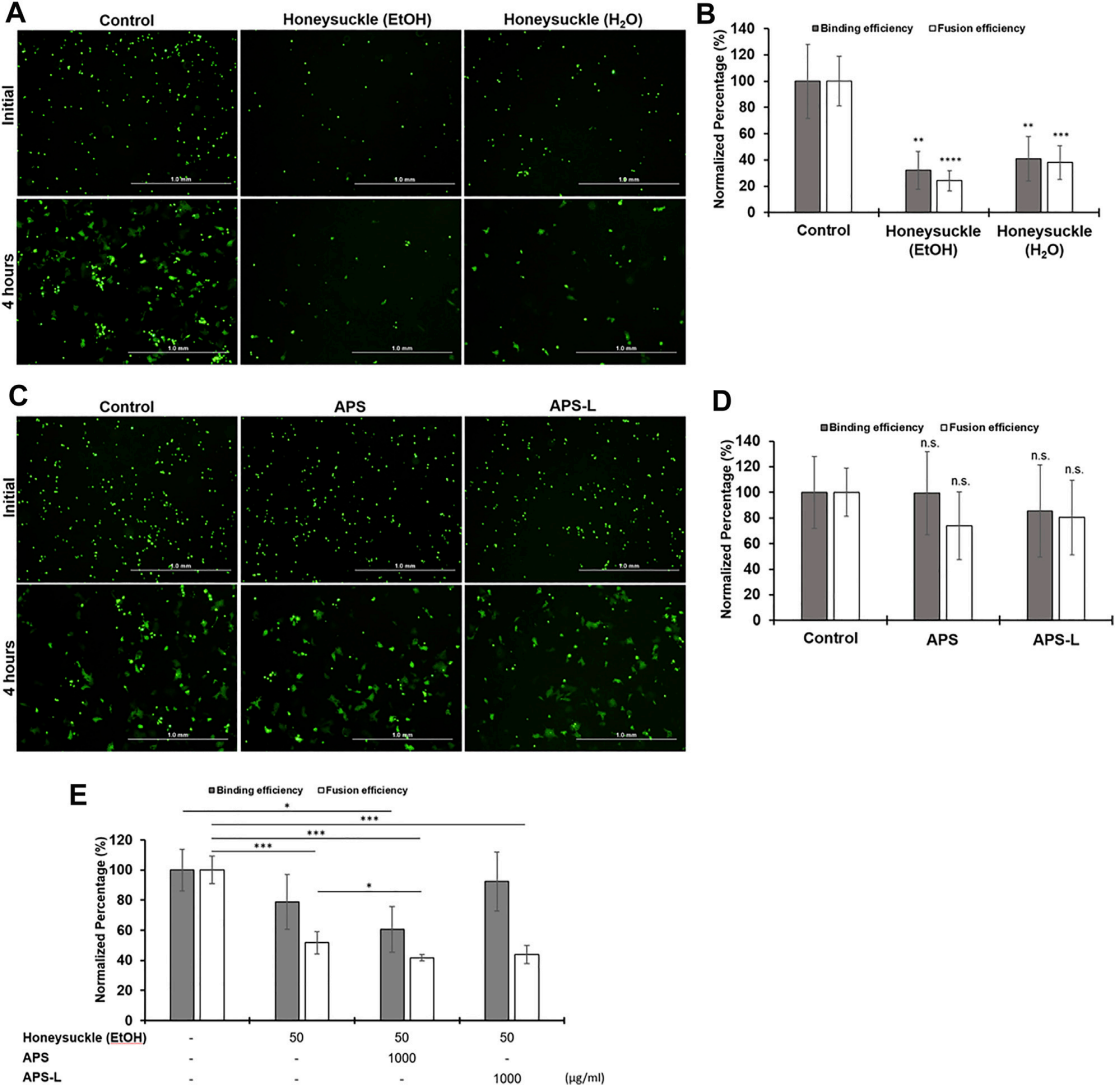
The effect of honeysuckle, Huangqi, and their combination on the binding of SARS-CoV2-spike with ACE2 and syncytia formation. **(A,C)** Imaging of EGFP/spike-positive BHK-21 effector cell binding (upper panel) and fusion (lower panel) with Calu-3 target cells in the absence or presence of honeysuckle **(A)**, Huangqi **(C)**, or combination of honeysuckle-EtOH and either APS or APS-L. The binding efficiency of SARS-CoV2-spike to ACE2 (gray bars) and the formation of syncytium indicating fusion efficiency (white bars) was quantified in the cells treated with honeysuckle **(B)**, Huangqi **(D)**, and honeysuckle-EtOH combined with either APS or APS-L **(E)**. *, *p* < 0.05; **, *p* < 0.01; ***, *p* < 0.001; n.s: no significance. Scale bar equals 1.0 mm in all figures.

The treatment of either honeysuckle-EtOH (500 μg/ml) or honeysuckle-H_2_O (500 μg/ml) resulted in a significant reduction of the number of EGFP-positive cells binding and syncytia formation (Figure 7A). The numbers of the initial fluorescent cells or the big fluorescent multinucleated cells with honeysuckle treatment at 4 h were smaller than that of the control, indicating the binding of SARS-CoV2 S protein with ACE2 receptor and syncytia formation were suppressed by honeysuckle. Honeysuckle-EtOH reduced protein binding and cell fusion to approximately 30 and 25% of the level of the control group, respectively, whereas both measurements were around 40% for the honeysuckle-H_2_O treatment group (Figure 7B). Notably, honeysuckle-EtOH showed higher suppression of protein binding and cell fusion compared to honeysuckle-H_2_O. In contrast, 1,000 μg/ml of APS or APS-L could not significantly suppress binding efficiency and syncytia formation compared to the control groups (Figures 7C,D). However, in the combination treatments, honeysuckle-EtOH (50 μg/ml) plus APS (1,000 μg/ml) could reduce 40% of the binding efficiency compared to the control. Moreover, honeysuckle-EtOH (50 μg/ml) combined with either APS (1,000 μg/ml) or APS-L (1,000 μg/ml) could significantly decrease the syncytia formation down to 50% (Figure 7E). These results suggested that honeysuckle alone or combined with Huangqi could act as an anti-SARS-CoV-2 agent by blocking SARS-CoV-2 S protein-related binding and fusion capability.

To examine whether honeysuckle and Huangqi might have the direct inhibitory effects of the binding between SARS-CoV-2 S protein and ACE2 by employing recombinant proteins, we investigated the inhibitory effect of Honeysuckle and Huangqi with the dose of 2, 4, and 8 mg/ml. Honeysuckle-EtOH could suppress the binding efficiency of trimeric spike protein from all five strains to ACE2 approximately 25–40% (Supplementary Figure S6). Honeysuckle-H_2_O could reduce the binding in wild-type strain by all three doses (Supplementary Figure S7A) and partially decline the attachment in alpha and gamma strain (Supplementary Figures S7B,E); however, honeysuckle-H_2_O could not affect beta and delta variant (Supplementary Figures S7C,D). Similarly, APS could slightly repress the binding from wild-type, alpha, and gamma strain, but not beta and delta variant (Supplementary Figure S8). Whereas, APS-L showed better effect with the reduction of binding efficacy of wild-type, alpha, delta, and gamma reaching statistical analysis (Supplementary Figure S9). Among four tested drugs, honeysuckle-EtOH appeared to be the most effective, demonstrated by the high binding reduction and being the only treatment that was able to confront spike protein beta strain.

Given that honeysuckle-EtOH reduced protein binding and cell fusion, we also evaluated the expression of two key cellular factors, ACE2 and TMPRSS2, required for viral infection by western blot assay. The treatment of 500 μg/ml Honeysuckle-EtOH in Calu-3 cells caused the downregulation only in ACE2 expression, but not in TMPRSS2 expression (Supplementary Figure S10). This observation was correlated with the cell binding experiment results in Figure 7A.

## Discussion

Due to the high mutation rate of SARS-CoV-2, the strategy of targeting multiple mechanisms of a virus may increase a therapy’s efficacy against the emerging virus variants. Notably, asymptomatic patients with COVID-19, accounting for 40–45% of SARS-CoV-2 infections, can transmit the virus for longer than 14 days (Oran and Topol, 2020). In addition, severe COVID-19 patients experience cytokine storm-related symptoms that lead to fatal ARDS with an incident rate of 41.8% (Wu C et al., 2020). The patients who have recovered from ARDS remain at high risk of disease sequelae since the lungs cannot regenerate themselves. In worse cases, ARDS may progress to pulmonary fibrosis. Therefore, due to the emergence of the viral variants and the serious after-effects of the viral infection, there is an urgent demand for drugs that can suppress a broad spectrum of targets in the SARS-CoV-2-related mechanisms.

Here, we have comprehensively analyzed the current medications of COVID-19 and the viral infection mechanisms *via* evidence-based retrieval databases UpToDate (UpToDate, 1992) and DynaMed (DynaMed, 1995). This bioinformatics approach provides an effective screening method to predict the list of TCM candidates, covering a wide range of pharmacologic functions (such as antipyretics, antitussives, or antiasthmatics etc.) (Figure 1; Table 2), against SARS-CoV-2 infection. To further sort out highly potential TCM drugs capable of anti-SARS-CoV-2 activity from the list, SymMap (Wu et al., 2019) was chosen as the primary source since it integrated various databases of herbal ingredients and drug target databases. In order to optimize our prediction of TCMs, we chose ten target genes, which are most frequently targeted by over 200 TCMs. These target genes are: *TNF, CYP1A2, CYP3A4, NOS2, IL6, IL1B, STAT1, NFKB1A, CXCL10*, and *IFNG* (Figure 1D). Using these genes, we could find the connection with the drugs against COVID-19. For example, *TNF* linked to Chloroquine, *CYP1A2* linked to Ritonavir, *CYP3A4* linked to Dexamethasone and Ritonavir, and *NOS2* linked to Dexamethasone. In other word, only targets linked to Chloroquine, Ritonavir, and Dexamethasone had strong connection to our predicted TCM candidates. The mechanism of action (MoA) of the predicted TCMs, especially extracts from honeysuckle and Huangqi, may be different from these drugs since they were connected to very limited targets. To analyze MoA of specific drugs (either TCM, small molecule inhibitor, or miRNA) through target analysis, we should obtain the full set of targets from corresponding databases related to each compound (ex. Figures 2, 3). Some popular anti-SARS-CoV-2 drugs such as Remdesivir, Baricitinib, and Tocilizumab are not predicated in our TCM candidate list. Because we do not know their targets under current database researching (Table 1). Therefore, we need another target resource from COVID-19 related infection and ARDS mechanism to make more comprehensive prediction (Figure 1A).

Through our bioinformatics workflow (Figure 1), we discovered that honeysuckle participated in regulating several inflammatory pathways, including those involving macrophages, fibroblasts, glucocorticoid receptors, and IL-12 signaling (Figure 2D). In addition, Huangqi might be involved in pathways mimicking glucocorticoid function, such as pregnane X receptor/ retinoid X receptor (PXR/RXR) signaling and xenobiotic metabolism (Figure 2E) (Bertilsson et al., 1998; Kliewer et al., 2002). However, these analyses only provided the broad mechanisms of the TCM candidates without the specific knowledge of their effects on SARS-CoV-2 replication. To clarify the core molecular features of COVID-19, we analyzed the SARS-CoV-2 infection profile from GSE147507, which contained the fold-change of gene expression in Calu-3 cells (Supplementary Figure S3). Particularly, *TNF* and *IL6* were detected as the critical markers of SARS-CoV-2 infection, consistent with other report (Choudhary et al., 2021). In contrast, the level of IFNβ is not used as a severity marker (da Silva et al., 2021), though IFNB1 were found to be significant in GSE147507.

One possibility of our candidate TCM drugs might regulate TNF-α and IL-6 is via inducing host miRNA. This concept is also supported by our previous work (Lee et al., 2021). Because there is no database of herb-induced miRNAs, it is challenging to predict whether a TCM candidate can induce a specific miRNA. Moreover, the active ingredients of honeysuckle and Huangqi responsible for inducing various innate miRNAs or exerting anti-viral effects remain poorly understood. Thus, we indirectly integrated the information of the targets of TCM candidates and miRNAs *via* SymMap and miRDB that provided an online miRNA target mapping software. The predictions were successfully validated honeysuckle and Huangqi as inducers of the targeted miRNAs including *let-7a*, *miR-148b*, and *miR-146a*, which was consistent with our *in vitro* results in which honeysuckle and Huangqi upregulated the targeted miRNAs expression to different levels (Figure 4 and Supplementary Table S6), thereby suppressing SARS-CoV-2 replication.

The interaction between miRNA and its target mRNA leads to the degradation of bound mRNA, thereby repressing its replication. Such mechanisms are rationally applied for this particular SARS-CoV-2 infection, in which the viral genome is targeted and inhibited by a “matched” miRNA immediately upon its release from the nucleus of the host cell, resulting in the suppression of its multiplication and survival (Acuña et al., 2020). Innate immunity, which is the first defense barrier against foreign pathogens, triggers multiple inflammatory responses when it recognizes the invasion of a virus. Toll-like receptor (TLR), one of the recognition cascades, triggers various intercellular signaling *via* two possible pathways: MyD88 and Toll/IL-1R domain-containing adaptor-inducing IFN-β (TRIF) pathway, which subsequently activates NF-kB signaling pathway. The activation of this pathway results in the release of pro-inflammatory cytokines including IL-6 and TNF-α, which are vastly elevated ones in severe COVID-19 patients (Chandan et al., 2019). In addition, it has been reported that ORF3a, M, ORF7a, and N proteins of SARS-CoV-2 are NF-κB activators (Su et al., 2021). It has been reported that *miR-146a* could negatively regulate NF-κB pathway (Sheedy and O'Neill, 2008). A consistent decrease in *miR-146a-5p* levels was observed in COVID-19 patients (Tang et al., 2020; Zhang S et al., 2021) and linked to the severity of COVID-19-related inflammation (Sabbatinelli et al., 2021). Moreover, NF-κB pathway could also be repressed by *miR-148b* by targeting and inhibiting the MyD88 expression which is an immune generator, bridging extracellular signals (Chu et al., 2017). Our *in silico* analysis revealed that *miR-148b* might regulate TGF-ꞵ and CTLA4 signaling pathways, both of which were the important components of cellular immunity against viral infections (Figure 3B). Furthermore, *let-7a* could suppress the positive feedback loop between NF-κB and IL-6. Our bioinformatics analysis also reported that *let-7a* might mediate inflammation by regulating IL-6, MAPK, PI3K-Akt, and FoxO (Figure 3A and Supplementary Table S5). In the cell–cell fusion assay, we used S protein of the Wuhan strain for validation, and the results showed that both honeysuckle and Huangqi may inhibit the binding and fusion stage of SARS-CoV-2 infection. In the additional ELISA experiments, we examined the effects of our candidate TCM on interfering the binding of different strains of SARS-CoV-2 S proteins (Wuhan, α, β, γ, and δ) to biotinylated human ACE2. SARS-CoV-2 variants of interest (α, β, γ, and δ) were identified as early as September 2020, and they were classified according to the genetic diversity of receptor-binding domain (RBD) region on S protein, such mutations increase binding affinity of the S protein to ACE2, thus enhancing the viral attachment and entry into host cells (Aleem et al., 2021). Our results showed that honeysuckle-EtOH suppressed all strains of S protein-ACE2 binding; both honeysuckle-H_2_O and APS suppressed Wuhan, α, and γ strains; APS-L suppressed Wuhan, α, δ, and γ strains (Supplementary Figures S6–S9). The similar inhibition trend of honeysuckle-H_2_O and honeysuckle-EtOH were also observed in the ELISA based S protein and hACE2 binding assays (Supplementary Figures S6–S8). Compared to the binding inhibition of TCMs in the cell-cell fusion assays, however, TCMs caused less inhibitory effects in ELISA assays. It may be due to S proteins in ELISA assay that were purified recombinant proteins whose conformation and post-translation modification may be different from S proteins expressed in BHK-21 cells. In addition, the intersection of the significant targets among honeysuckle, Huangqi, *let-7a*, and *miR-148b* suggested that the most important target between TCM candidates and miRNAs might be *IL6* (Figures 3C,D), suggesting that inducing these targeted miRNAs might decrease the level of pro-inflammatory cytokines involving in cytokine storm and ARDS. Thus, we examined the efficacy of our TCM candidates on reducing cytokine storms. Although not all of the predicted cytokines (Supplementary Figure S4) were decreased, honeysuckle and Huangqi substantially complemented the immunomodulatory function against SARS-CoV-2 infection by suppressing IL-6 and TNF-α, respectively (Figure 5), consistent with our bioinformatics analysis above. Taken together, these results suggested that both honeysuckle and Huangqi could not only increase the expression of *let-7a*, *miR-148b*, and *miR-146a* to different levels to effectively block viral replication but also inhibit cytokine storm by reducing IL-6 and TNF-α release level, respectively. Hence, the combination therapy of honeysuckle and Huangqi might be complementary to each other and improved COVID-19 treatment. Alternatively, we could use other TLR and cytokine stimulations, which are more representative to SARS-CoV-2 infection for this experiment.

The host cell invasion of SARS-CoV-2 starts with the binding of viral spike protein to the human ACE2 receptor, enabled by the cleavage of the receptor-binding domain (RBD) region on S1 from the spike protein during viral binding and entry procedure (Samavati and Uhal, 2020). This process of accessing host cells, dependent on the interaction between the complex sugar molecules (glycans) on the surface of viruses and host cells via glycoproteins, is required for SARS-CoV-2 replication (Huang et al., 2020). Glycans found on the spike protein are only marginally involved in the binding of the virus to human cells; however, they are vital in the virus’ fusion with the host cell and cell entry (Suzuki et al., 2021). Since Huangqi contains polysaccharides, we hypothesized that Huangqi might compete with the glycans on the spike proteins; which could explain the role of Huangqi in blocking fusion despite its statistically insignificant data (Figures 7C,D). Meanwhile, both honeysuckle-H_2_O and honeysuckle-EtOH have demonstrated their remarkable ability to prevent the attachment of viral spike protein and the ACE2 receptor and their formation of syncytium. These data suggested that these treatments could negatively regulate not only glycans but also the RBD, reducing the docking of the virus on host ACE2 and viral entry. In addition, honeysuckle-EtOH could support APS and APS-L efficacy in inhibiting the binding of spike protein and ACE2 receptor and their fusion in combination treatments. Therefore, it is essential to investigate further mechanisms of those treatments on spike glycans and whether it is feasible to bind to glycans on ACE2; these experiments can be useful in enhancing our understanding of different virus variants. In addition, both honeysuckle-EtOH and APS-L could suppress M^pro^ activity in a dose-dependent manner (Figures 6B,C), indicating that these two herbs could inhibit SARS-CoV-2 replication. Furthermore, honeysuckle-EtOH was more effective than APS-L, possibly because it contained luteolin, a promising M^pro^ antagonist (Shawan et al., 2021). These results may help explain the significant inhibitory effect of honeysuckle on viral fusion and host cell entry.

It is common in the clinical practice of TCM to prescribe a combination of different herbs or formulas to treat various symptoms (Yeh et al., 2014). Honeysuckle is widely used in China, Korea, and Japan. The putative active ingredients in honeysuckle flower buds include luteolin, chlorogenic acid, linalool, isochlorogenic acid, and shuangkangu (Lee et al., 2017). Honeysuckle is an essential ingredient in more than half of the patented anti-inflammatory TCM drugs, and it is one of the most prescribed herbs in the treatment or prevention of COVID-19 (Li J et al., 2020; Luo et al., 2020). For example, honeysuckle is the main component of honeysuckle decoction*,* Lianhuaqingwen Capsule, and Shuang-Huang-Lian oral solution. All of the above-patented formulas can potentially target SARS-CoV-2 infection or the critical proteins in virus-induced cytokine storms, such as TNF-α, IL-1β, and IL-6 (Zhou et al., 2020; Zhang FX et al., 2021; Liu et al., 2021). The complexity of natural products makes it difficult to conclude a specific ingredient to explain all MoA of a TCM, therefore a detained and standardized HPLC fingerprint may help to address this issue. Our honeysuckle sample contained relatively abundant chlorogenic acid, cynaroside, and 3,5-dicaffeoylquinic acid. Chlorogenic acid is one of the most important bioactive ingredients of honeysuckle, and it exerts remarkable anti-SARS-CoV-2 activity according to other researches (Yu et al., 2020; El Gizawy et al., 2021; Wang et al., 2021). The main potential targets of chlorogenic acid investigated by molecular docking include IL6 and ACE (Wang et al., 2021), both of which help to explain the results of our *in vitro* cytokine assays and cell-cell fusion assays. Cynaroside is also known as luteoloside, and it is a strong inhibitor of methyltranferase of SARS-CoV-2 (Chandra et al., 2021). 3,5-dicaffeoylquinic acid may act as an inhibitor of SARS-CoV-2 spike RBD, which is a crucial protein for viral entry (Singh et al., 2021).

Huangqi, widely used to enhance the immune system, contains polysaccharides, saponins, flavonoids, linoleic acid, and alkaloids. Huangqi can be used as a crude extract or APS, the most critical active component in Huangqi. APS regulates immune functions by stimulating the release of cytokines and affecting the secretion of immunoglobulin and conduction of immune signals. The immunomodulatory effects of APS against various viruses have been demonstrated (Shi et al., 2014; Xue et al., 2015; Wang et al., 2016; Zheng et al., 2020). APS has also been investigated for its enhancement of the immunity of COVID-19 patients (Adhikari et al., 2020; Meletis and Wilkes, 2020).

For a TCM clinician, a combination of honeysuckle and APS may have a synergistic influence in clearing the heat toxin (anti-inflammation) and tonifying qi (immune support and modulation), the latter of which is a therapeutic method to replenish physical strength and treat qi deficiency (WHO, 2007). Meanwhile, for a physician of infectious disease, TCM combination therapy may induce many anti-viral miRNAs, thus suppressing SARS-CoV-2 replication and subsequent transmission. Further investigations on the effect of the honeysuckle-APS combination on COVID-19 patients are needed.

Taken together, novel TCM candidates could be prioritized through *in silico* predictions, followed by validation using various anti-viral activity assays. This study highlights two conclusions—first, honeysuckle and Huangqi exhibit diverse but intimately complementary anti-SARS-CoV2 activities. Second, systems biology-based drug screening via integrative data mining strategy is not only highly valuable in identifying and repurposing TCM drugs, but also in unveiling innovative potential for future anti-viral drug development.

## Data Availability

Publicly available datasets were analyzed in this study, and we took 3 repeated results of Calu3 cells from a total of 110 samples. This data can be found on the GEO database: https://www.ncbi.nlm.nih.gov/geo/query/acc.cgi?acc=GSE147507.
