# Supplementary material for: Honeysuckle (Lonicera japonica) and Huangqi (Astragalus membranaceus) Suppress SARS-CoV-2 Entry and COVID-19 Related Cytokine Storm in Vitro
Source: Front Pharmacol. 2022 Mar 25;12:765553. doi: 10.3389/fphar.2021.765553 (PMC8990830; doi:10.3389/fphar.2021.765553)
Supplement: Supplementary file 1 [file DataSheet1.PDF]

Supplementary Figures

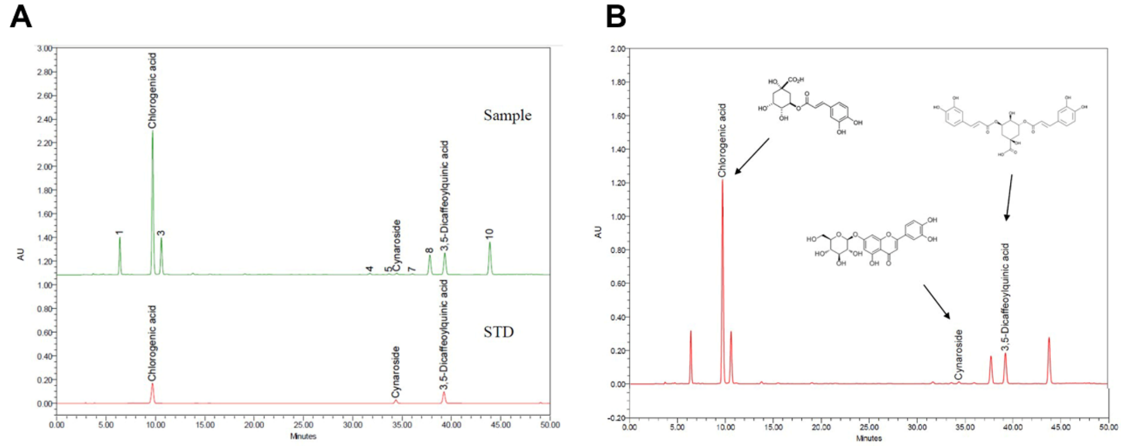

Supplementary Figure 1. HPLC fingerprinting profiles of Honeysuckle-H_2O extract.

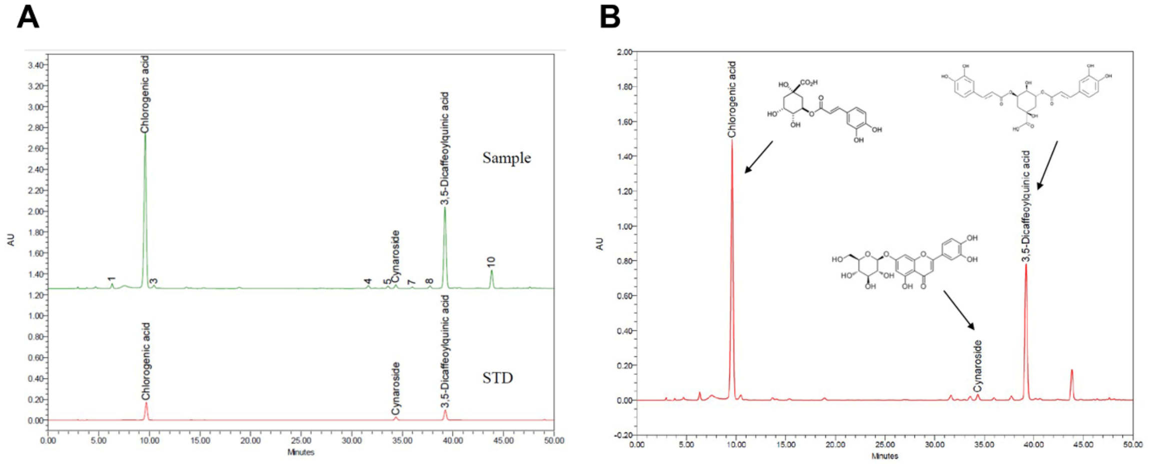

Supplementary Figure 2. HPLC fingerprinting profiles of Honeysuckle-EtOH extract.

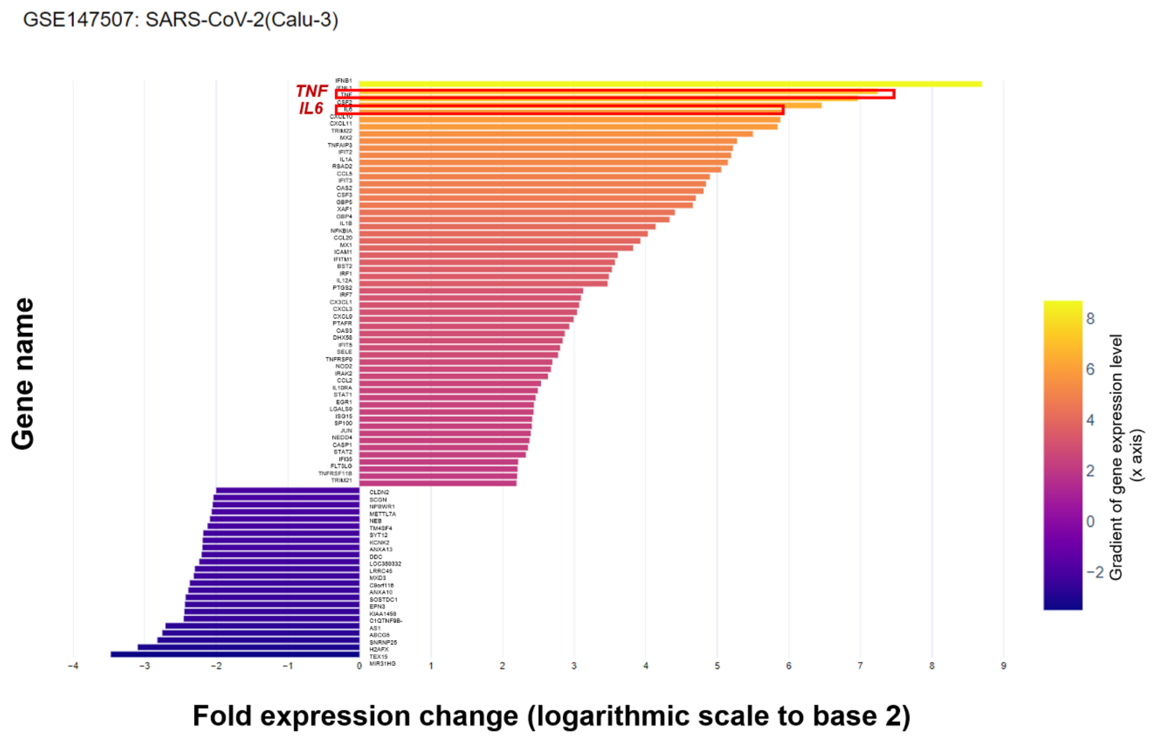

Supplementary Figure 3. The differential expression genes in Calu-3 cells infected with SARS-CoV-2. The gene expression profile of SARS-CoV-2 infection in Calu-3 (a lung adenocarcinoma) cell (GSE147507) was retrieved from the GEO database. The y-axis indicates the gene names, and the x-axis indicates log2 fold-change. *TNF* and *IL6* were upregulated.

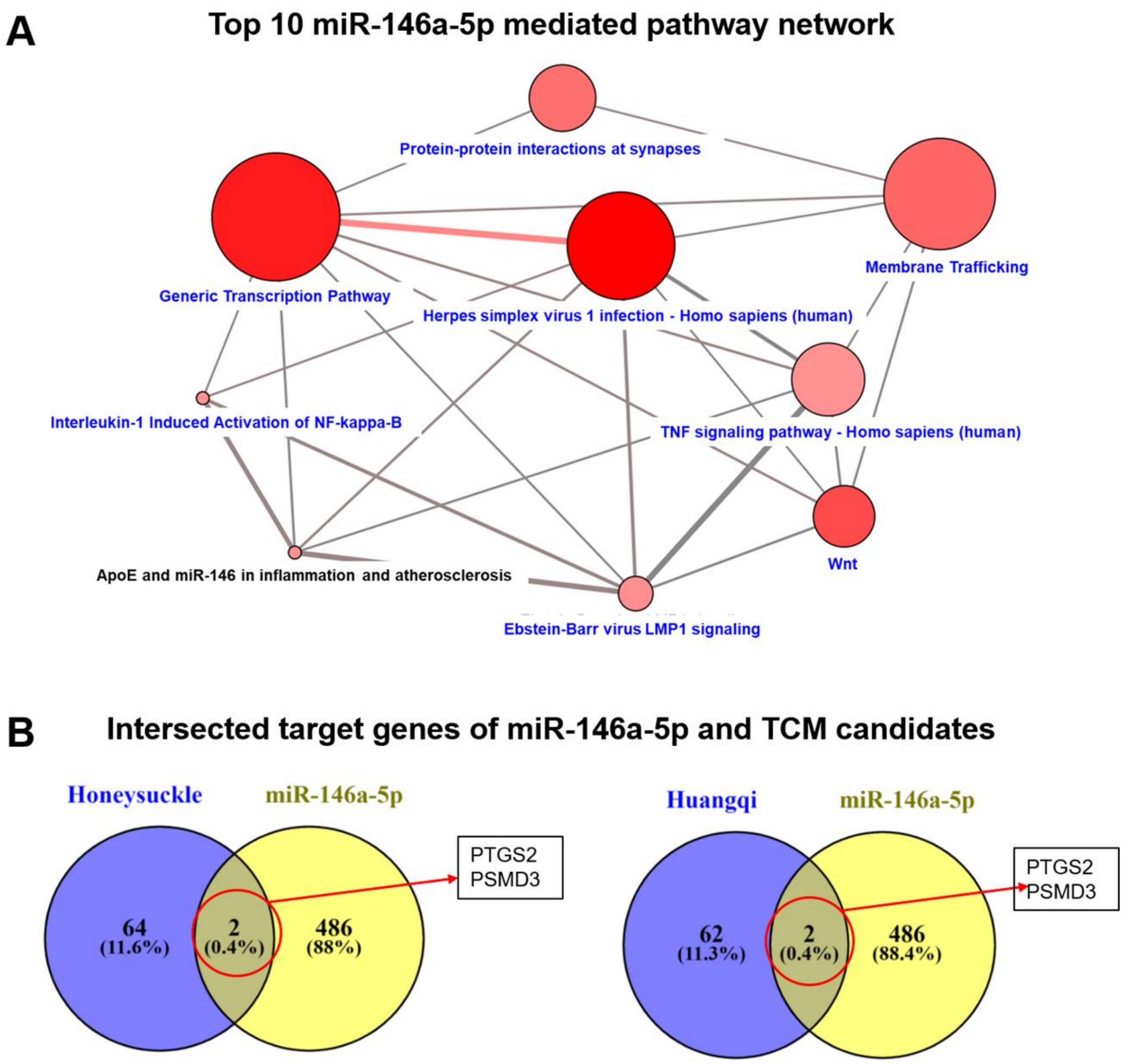

Supplementary Figure 4. Multi-database analysis of the functions of *miR-146a* and its connections to the TCM candidates. miRNA-related targets were obtained from miRDB, and each list of genes (488 target genes from *miR-146a-5p*) was used to query CPDB for signaling pathways. (A) The respective network of the 10 signaling pathways related to this study and mediated by *miR-146a-5p*. Pathways in black are highly related to this study. (B) The intersected target genes of miRNA and TCM candidates. Both honeysuckle and Huangqi had common targets, includingPTGS2 and PSMD3 genes, with *miR-146a-5p*.

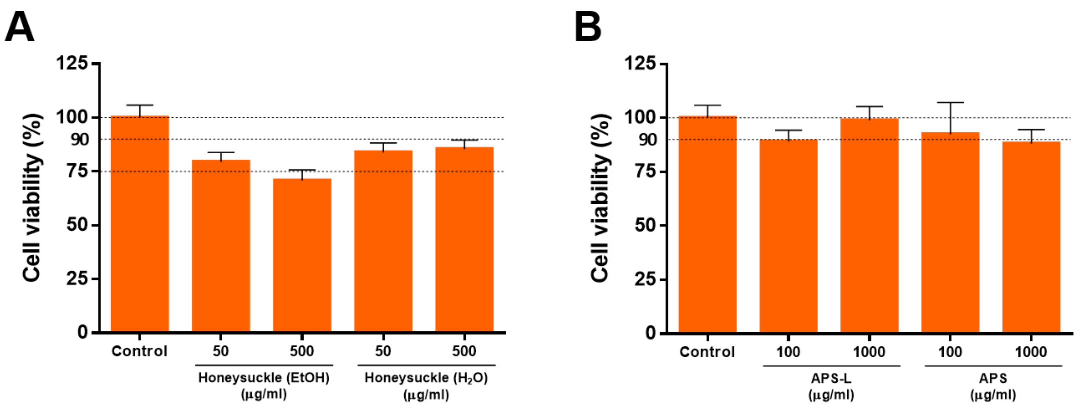

Supplementary Figure 5. BEAS2B cell viability after honeysuckle and APS treatment.

After 24 h of treatment with (A) honeysuckle-EtOH or honeysuckle-H_2O and (B) APS-L or APS on BEAS2B cells, cell viability was measured using the SRB assay.

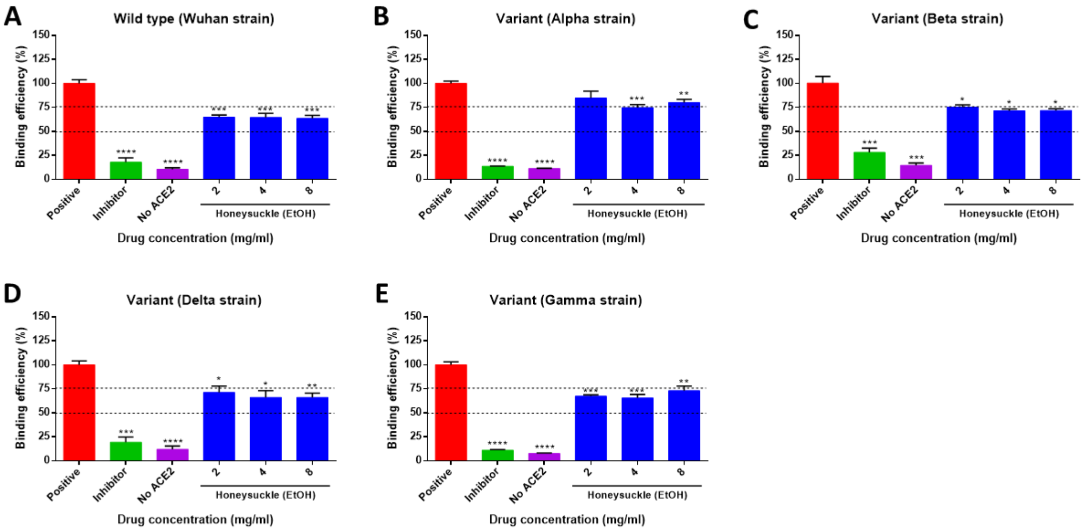

Supplementary Figure 6. Variant specificity of Honeysuckle (EtOH) against the trimeric spike protein binding to ACE2. Trimeric spike proteins derived from (A) wild-type/Wuhan strain, (B) variant alpha, (C) variant beta, (D) variant delta, and (E) variant gamma were used for ELISA based spike protein and hACE2 binding assays. A positive condition represents the full binding activity of trimeric spike protein on hACE2. The inhibitor used was the RBD antibody (10 μg/mL). Data represent Mean ± SEM (n = 4). A *, **, or *** indicates a significant difference to the corresponding control sample with p < 0.05, 0.01, or 0.001, respectively when compared to the binding efficiency of the positive group.

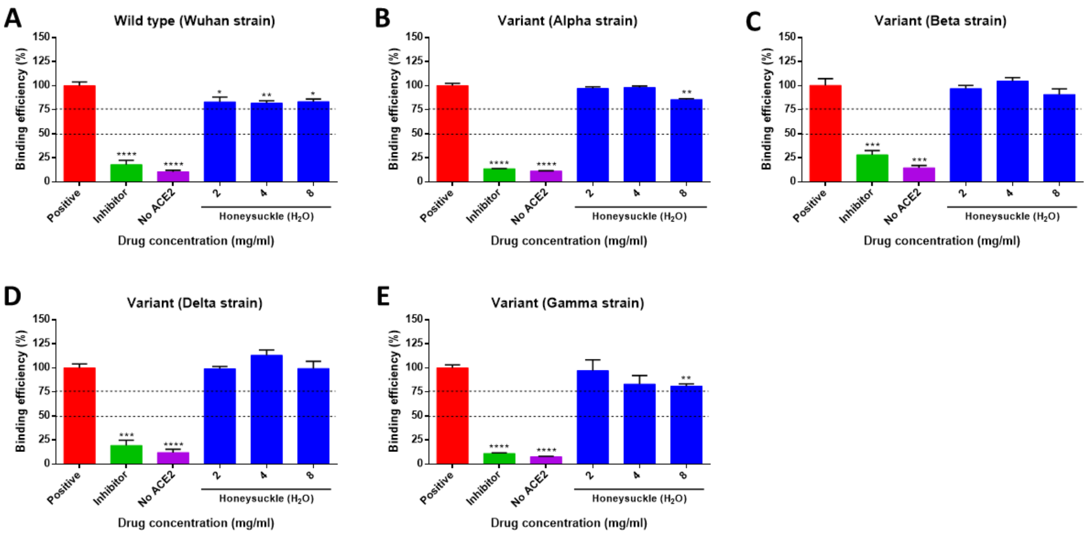

Supplementary Figure 7. Variant specificity of Honeysuckle (H_2O) against the trimeric spike protein binding to ACE2. Trimeric spike proteins derived from (A) wild-type/Wuhan strain, (B) variant alpha, (C) variant beta, (D) variant delta, and (E) variant gamma were used for ELISA based spike protein and hACE2 binding assays. A positive condition represents the full binding activity of trimeric spike protein on hACE2. The inhibitor used was the RBD antibody (10 μg/mL). Data represent Mean ± SEM (n = 4). A *, **, or *** indicates a significant difference to the corresponding control sample with p < 0.05, 0.01, or 0.001, respectively when compared to the binding efficiency of the positive group.

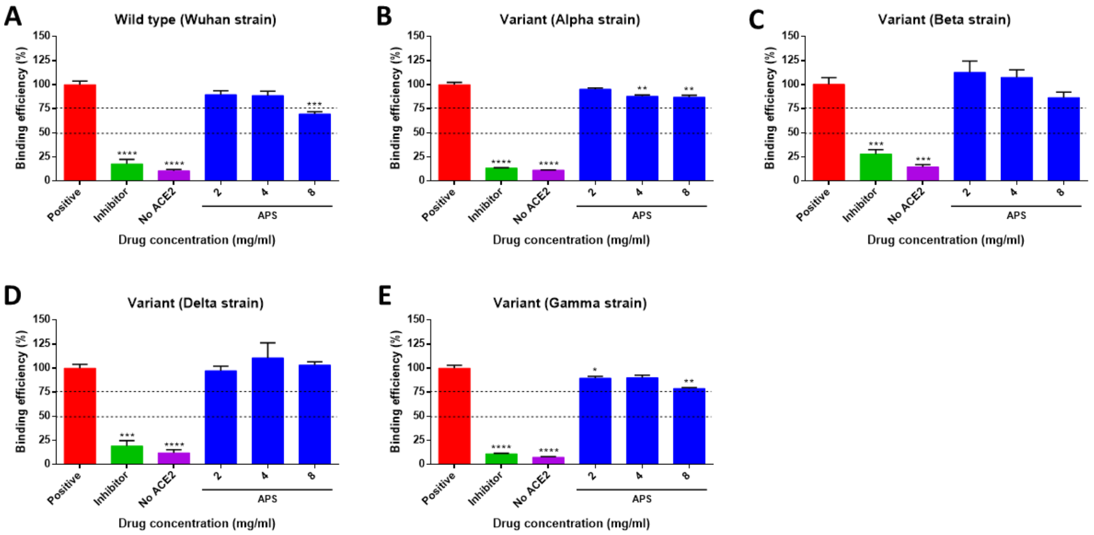

Supplementary Figure 8. Variant specificity of APS against the trimeric spike protein binding to ACE2. Trimeric spike proteins derived from (A) wild-type/Wuhan strain, (B) variant alpha, (C) variant beta, (D) variant delta, and (E) variant gamma were used for ELISA based spike protein and hACE2 binding assays. A positive condition represents the full binding activity of trimeric spike protein on hACE2. The inhibitor used was the RBD antibody (10 μg/mL). Data represent Mean ± SEM (n = 4). A *, **, or *** indicates a significant difference to the corresponding control sample with $p < 0.05$, 0.01, or 0.001, respectively when compared to the binding efficiency of the positive group.

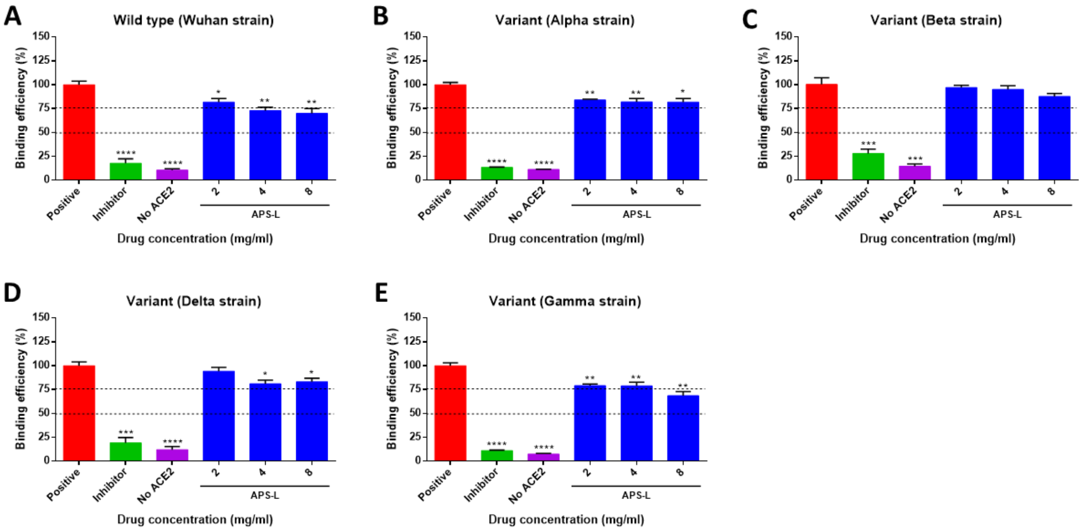

Supplementary Figure 9. Variant specificity of APS-L against the trimeric spike protein binding to ACE2. Trimeric spike proteins derived from (A) wild-type/Wuhan strain, (B) variant alpha, (C) variant beta, (D) variant delta, and (E) variant gamma were used for ELISA based spike protein and hACE2 binding assays. A positive condition represents the full binding activity of trimeric spike protein on hACE2. The inhibitor used was the RBD antibody (10 μg/mL). Data represent Mean ± SEM (n = 4). A *, **, or *** indicates a significant difference to the corresponding control sample with $p < 0.05$, 0.01, or 0.001, respectively when compared to the binding efficiency of the positive group.

Given that honeysuckle-EtOH reduced protein binding and cell fusion, we also evaluated the expression of two key cellular factors, ACE2 and TMPRSS2, required for viral infection by western blot assay. The treatment of 500 μg/ml Honeysuckle-EtOH in Calu-3 cells caused the downregulation only in ACE2 expression, but not in TMPRSS2 expression (Supplementary Figure 10). This result was correlated with the cell-binding experiment data in Figure 7A.

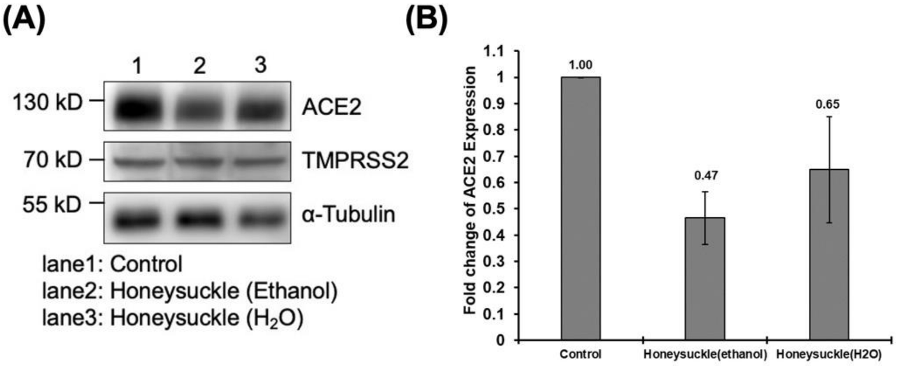

Supplementary Figure 10. Honeysuckle-EtOH down-regulates ACE2 expression. Calu-3 cells were incubated with 500 μg/ml Honeysuckle (ethanol) or Honeysuckle (H_2O) for 5 hours. The expression levels of ACE2 and TMPRSS2 in Calu-3 cells were detected by Western blotting and normalized by α-Tubulin expression. The number of biological replicates was 2.

Supplementary Table

Supplementary Table 1. The number of connected TCM candidates according to different drug target genes.

Genetic targets of current medication	No. of connected TCM *
TNF	**441**
NOS2	**328**
CYP1A2	**208**
CYP3A4	**203**
NR3C1	98
CHRNA7	91
PLA2G1B	86
SLC6A4	77
GSTA2	60
NR3C2	59
CYP2B6	41
HRH2	16
GLRA3	15
HSD11B1	9
TLR9	9
ANXA1	9
CYP2C19	8
CYP2C8	7
CYP3A7	7
P2RX7	7
CYP3A5	6
MLNR	6
SMO	4
TLR7	4
GABRB3	3
NR0B1	3
SERPINA6	3
PLA2G1B	N/A
TLR9	N/A
MRGPRX1	N/A

* Filtered by FDR-BH < 0.05.

*N/A: not available in SymMap database

Bold: only number of connected TCM > 200 are taken into accounts.

Supplementary Table 2. Number of connected TCM candidates revealed by using various ARDS therapeutic target genes.

ARDS targets	No. of connected TCM *
TNFA (TNF)	**441**
IL6	**282**
IL1B	**279**
STAT1	**230**
NFKBIA	**228**
CXCL10	**222**
IFNG	**214**
NFKB1	140
STAT3	103
TLR4	66
ACE	54
STAT4	53
IFNB1	43
IFNAR2	43
JAK2	30
IL2RA	29
Bcl3	28
JAK1	25
ACE2	21
NFKBIB	21
AGTR1	20
NFKB2	20
NFKBIE	20
NLRP3	19
IL7R	17
STAT5A	12
JAK3	11
IL17B	11
IL17F	9
IFNA13	8
STAT5B	8
STAT6	8
AGTR2	6
STAT2	4
TLR7	4
TLR8	4
IL17RA	4

IFNGR2	4
IL2RB	3
IL2RG	3
TYK2	3
TLR3	1
IL8	1
IL17RC	0
IL17RD	0
IFNGR1	0
NFKBIC	N/A
P100	N/A
P105	N/A

* Filtered by FDR-BH < 0.05.

*N/A: not available in SymMap database

Bold: only number of connected TCM > 200 are taken into accounts.

Supplementary Table 3. Top 50 Honeysuckle and Huangqi mediated pathways analyzed by CPDB.

A

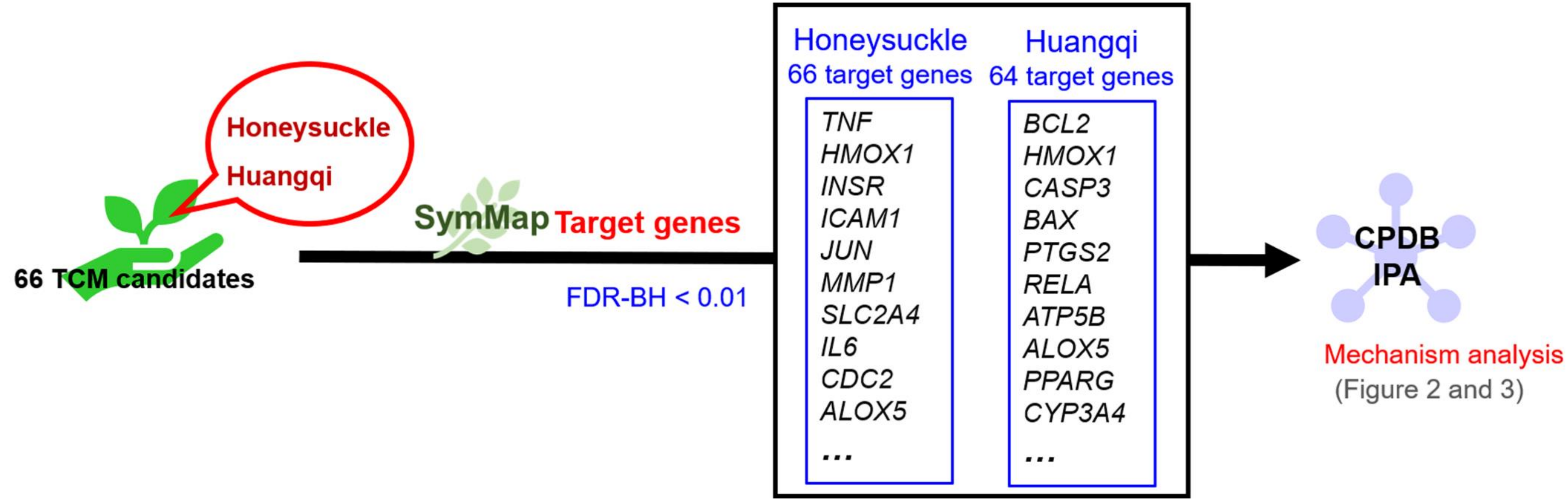

We selected honeysuckle and Huangqi from 66 TCM candidates for bioinformatics analysis. Honeysuckle and Huangqi had 66 target genes and 64 target genes, respectively, and these were retrieved from SymMap with FDR-BH < 0.01.

B

Rank	Honeysuckle mediated pathway	p-value	q-value	Pathway source
1	Pathways in cancer - Homo sapiens (human)	4.45E-23	4.77E-20	KEGG
2	Human cytomegalovirus infection - Homo sapiens (human)	2.93E-22	1.57E-19	KEGG
3	Lipid and atherosclerosis - Homo sapiens (human)	3.67E-21	1.31E-18	KEGG

4	AGE-RAGE signaling pathway in diabetic complications - Homo sapiens (human)	7.00E-21	1.88E-18	KEGG
5	Kaposi sarcoma-associated herpesvirus infection - Homo sapiens (human)	1.41E-20	3.01E-18	KEGG
6	Spinal Cord Injury	9.90E-20	1.77E-17	Wikipathways
7	IL-18 signaling pathway	4.77E-19	7.30E-17	Wikipathways
8	Hepatitis B - Homo sapiens (human)	6.45E-19	8.64E-17	KEGG
9	Epstein-Barr virus infection - Homo sapiens (human)	9.39E-19	1.12E-16	KEGG
10	Photodynamic therapy-induced NF-kB survival signaling	3.68E-18	3.94E-16	Wikipathways
11	Hepatitis C and Hepatocellular Carcinoma	1.78E-17	1.73E-15	Wikipathways
12	Glucocorticoid receptor regulatory network	3.53E-17	3.15E-15	PID
13	Small cell lung cancer - Homo sapiens (human)	2.40E-16	1.98E-14	KEGG
14	Small cell lung cancer	4.27E-16	3.27E-14	Wikipathways
15	African trypanosomiasis - Homo sapiens (human)	5.63E-16	4.02E-14	KEGG
16	cadmium induces dna synthesis and proliferation in macrophages	1.84E-15	1.20E-13	BioCarta
17	Measles - Homo sapiens (human)	2.01E-15	1.20E-13	KEGG
18	Fluid shear stress and atherosclerosis - Homo sapiens (human)	2.01E-15	1.20E-13	KEGG
19	Human T-cell leukemia virus 1 infection - Homo sapiens (human)	2.67E-15	1.48E-13	KEGG
20	ATF-2 transcription factor network	2.76E-15	1.48E-13	PID
21	Gastrin signaling pathway	4.31E-15	2.20E-13	Wikipathways
22	T-Cell antigen Receptor (TCR) pathway during Staphylococcus aureus infection	4.91E-15	2.39E-13	Wikipathways
23	Hepatitis B infection	7.13E-15	3.32E-13	Wikipathways
24	Aryl Hydrocarbon Receptor Netpath	8.70E-15	3.89E-13	Wikipathways
25	PI3K-Akt signaling pathway	1.01E-14	4.35E-13	Wikipathways
26	Hepatitis C - Homo sapiens (human)	1.13E-14	4.64E-13	KEGG
27	IL-17 signaling pathway - Homo sapiens (human)	1.33E-14	5.28E-13	KEGG
28	AP-1 transcription factor network	1.68E-14	6.42E-13	PID
29	Calcineurin-regulated NFAT-dependent transcription in lymphocytes	1.74E-14	6.42E-13	PID

30	PI3K-Akt signaling pathway - Homo sapiens (human)	2.05E-14	7.31E-13	KEGG
31	Non-small cell lung cancer	2.33E-14	8.07E-13	Wikipathways
32	Chromosomal and microsatellite instability in colorectal cancer	2.74E-14	9.17E-13	Wikipathways
33	Netrin-UNC5B signaling pathway	3.31E-14	1.07E-12	Wikipathways
34	Leishmaniasis - Homo sapiens (human)	4.36E-14	1.37E-12	KEGG
35	T cell receptor signaling pathway - Homo sapiens (human)	4.65E-14	1.42E-12	KEGG
36	Nuclear Receptors Meta-Pathway	5.40E-14	1.61E-12	Wikipathways
37	HIF-1 signaling pathway - Homo sapiens (human)	8.28E-14	2.40E-12	KEGG
38	TNF signaling pathway - Homo sapiens (human)	1.15E-13	3.26E-12	KEGG
39	Apoptosis	2.05E-13	5.63E-12	Wikipathways
40	Oncostatin M Signaling Pathway	3.57E-13	9.57E-12	Wikipathways
41	DNA damage response	5.74E-13	1.50E-11	Wikipathways
42	MicroRNAs in cancer - Homo sapiens (human)	6.05E-13	1.55E-11	KEGG
43	Prostate cancer - Homo sapiens (human)	7.03E-13	1.75E-11	KEGG
44	Human immunodeficiency virus 1 infection - Homo sapiens (human)	7.31E-13	1.78E-11	KEGG
45	Influenza A - Homo sapiens (human)	9.45E-13	2.25E-11	KEGG
46	Non-small cell lung cancer - Homo sapiens (human)	1.04E-12	2.43E-11	KEGG
47	Amoebiasis - Homo sapiens (human)	1.11E-12	2.52E-11	KEGG
48	mechanism of gene regulation by peroxisome proliferators via ppara	1.90E-12	4.25E-11	BioCarta
49	keratinocyte differentiation	2.28E-12	4.99E-11	BioCarta
50	Acute viral myocarditis	2.39E-12	5.13E-11	Wikipathways

C

**Rank**	**Huangqi mediated pathway**	**p-value**	**q-value**	**Pathway source**
1	Pathways in cancer - Homo sapiens (human)	3.28E-22	3.25E-19	KEGG
2	Interleukin-4 and Interleukin-13 signaling	1.64E-19	8.10E-17	Wikipathways

3	Fluid shear stress and atherosclerosis - Homo sapiens (human)	1.22E-18	4.03E-16	KEGG
4	AGE-RAGE pathway	1.84E-18	4.56E-16	Wikipathways
5	Selenium Micronutrient Network	4.50E-17	8.90E-15	Wikipathways
6	Aryl Hydrocarbon Receptor	9.11E-17	1.50E-14	Wikipathways
7	AGE-RAGE signaling pathway in diabetic complications - Homo sapiens (human)	4.98E-16	7.04E-14	KEGG
8	Hepatitis B - Homo sapiens (human)	2.49E-15	3.08E-13	KEGG
9	Kaposi sarcoma-associated herpesvirus infection - Homo sapiens (human)	3.73E-15	4.10E-13	KEGG
10	Small cell lung cancer - Homo sapiens (human)	9.19E-15	9.09E-13	KEGG
11	Epstein-Barr virus infection - Homo sapiens (human)	1.10E-14	9.92E-13	KEGG
12	Prolactin Signaling Pathway	3.52E-14	2.91E-12	Wikipathways
13	Hepatitis C and Hepatocellular Carcinoma	6.07E-14	4.62E-12	Wikipathways
14	Prostate cancer - Homo sapiens (human)	5.69E-13	4.02E-11	KEGG
15	HIF-1 signaling pathway - Homo sapiens (human)	8.02E-13	5.29E-11	KEGG
16	Overview of nanoparticle effects	1.51E-12	8.83E-11	Wikipathways
17	Viral Acute Myocarditis	1.52E-12	8.83E-11	Wikipathways
18	Glucocorticoid receptor regulatory network	2.58E-12	1.42E-10	PID
19	Photodynamic therapy-induced NF-kB survival signaling	2.72E-12	1.42E-10	Wikipathways
20	Apoptosis	6.11E-12	3.03E-10	Wikipathways
21	Nuclear Receptors Meta-Pathway	8.77E-12	4.13E-10	Wikipathways
22	Corticotropin-releasing hormone signaling pathway	1.08E-11	4.87E-10	Wikipathways
23	Human cytomegalovirus infection - Homo sapiens (human)	2.24E-11	9.65E-10	KEGG
24	Tuberculosis - Homo sapiens (human)	2.38E-11	9.82E-10	KEGG
25	Prolactin	2.67E-11	1.06E-09	NetPath
26	Chromosomal and microsatellite instability in colorectal cancer	3.94E-11	1.47E-09	Wikipathways
27	VEGFA-VEGFR2 Signaling Pathway	4.08E-11	1.47E-09	Wikipathways
28	Ceramide signaling pathway	4.16E-11	1.47E-09	PID

29	Insulin resistance - Homo sapiens (human)	5.00E-11	1.71E-09	KEGG
30	Leptin signaling pathway	5.73E-11	1.89E-09	Wikipathways
31	TNF signaling pathway - Homo sapiens (human)	6.61E-11	2.04E-09	KEGG
32	DNA Damage Response (only ATM dependent)	6.61E-11	2.04E-09	Wikipathways
33	Toxoplasmosis - Homo sapiens (human)	8.66E-11	2.60E-09	KEGG
34	Hepatitis C - Homo sapiens (human)	1.01E-10	2.86E-09	KEGG
35	Nonalcoholic fatty liver disease	1.01E-10	2.86E-09	Wikipathways
36	Amyotrophic lateral sclerosis (ALS)	1.90E-10	5.22E-09	Wikipathways
37	Influenza A - Homo sapiens (human)	2.91E-10	7.80E-09	KEGG
38	IL-17 signaling pathway - Homo sapiens (human)	3.64E-10	9.48E-09	KEGG
39	PI3K-Akt signaling pathway - Homo sapiens (human)	5.17E-10	1.28E-08	KEGG
40	Oncostatin M Signaling Pathway	5.24E-10	1.28E-08	Wikipathways
41	Doxorubicin Pathway, Pharmacokinetics	5.29E-10	1.28E-08	PharmGKB
42	Apoptosis - Homo sapiens (human)	5.46E-10	1.29E-08	KEGG
43	Integrated Breast Cancer Pathway	5.94E-10	1.34E-08	Wikipathways
44	Folate Metabolism	5.94E-10	1.34E-08	Wikipathways
45	TNF related weak inducer of apoptosis (TWEAK) Signaling Pathway	6.21E-10	1.37E-08	Wikipathways
46	TNF	6.73E-10	1.45E-08	INOH
47	RAC1-PAK1-p38-MMP2 Pathway	7.60E-10	1.60E-08	Wikipathways
48	C-type lectin receptor signaling pathway - Homo sapiens (human)	1.00E-09	2.06E-08	KEGG
49	Non-alcoholic fatty liver disease (NAFLD) - Homo sapiens (human)	1.34E-09	2.69E-08	KEGG
50	Leishmaniasis - Homo sapiens (human)	1.36E-09	2.69E-08	KEGG

Supplementary Table 4. Honeysuckle and Huangqi-elicited pathways according to the IPA analysis.

A

Ingenuity Canonical Pathways-Honeysuckle	-log (p-value)
Systemic Lupus Erythematosus In B Cell Signaling Pathway	23.4
Aryl Hydrocarbon Receptor Signaling	22.2
Role of Macrophages, Fibroblasts and Endothelial Cells in Rheumatoid Arthritis	20.7
Hepatic Fibrosis Signaling Pathway	19.1
Glucocorticoid Receptor Signaling	18.4
Neuroinflammation Signaling Pathway	18
Colorectal Cancer Metastasis Signaling	17.8
IL-12 Signaling and Production in Macrophages	17.4
Role of Osteoblasts, Osteoclasts and Chondrocytes in Rheumatoid Arthritis	17.2
Molecular Mechanisms of Cancer	17.2

B

Ingenuity Canonical Pathways-Huangqi	-log (p-value)
Aryl Hydrocarbon Receptor Signaling	20.7
Hepatic Fibrosis Signaling Pathway	16.6
Neuroinflammation Signaling Pathway	15.4
Colorectal Cancer Metastasis Signaling	15.1
Xenobiotic Metabolism Signaling	14.5
PXR/RXR Activation	14.4
Sirtuin Signaling Pathway	14.2
Role of Macrophages, Fibroblasts and Endothelial Cells in Rheumatoid Arthritis	13.8
Glucocorticoid Receptor Signaling	13.1
Small Cell Lung Cancer Signaling	12

Supplementary Table 5. miRNA-mediated pathways according to the CPDB analysis.

A

Rank	*let-7a-5p* mediated pathway	p-value	q-value	pathway source
1	Angiopoietin Like Protein 8 Regulatory Pathway	1.00E-06	1.92E-03	Wikipathways
2	AGE-RAGE signaling pathway in diabetic complications - Homo sapiens (human)	3.47E-06	3.32E-03	KEGG
3	Hepatitis C and Hepatocellular Carcinoma	2.42E-05	1.39E-02	Wikipathways
4	Scavenging by Class A Receptors	2.91E-05	1.39E-02	Reactome
5	FoxO signaling pathway - Homo sapiens (human)	4.63E-05	1.77E-02	KEGG
6	Collagen formation	6.52E-05	1.85E-02	Reactome
7	Collagen chain trimerization	6.76E-05	1.85E-02	Reactome
8	Insulin Signaling	8.44E-05	1.93E-02	Wikipathways
9	EGF-Core	9.97E-05	1.93E-02	Signalink
10	Type II diabetes mellitus - Homo sapiens (human)	1.01E-04	1.93E-02	KEGG
11	Insulin-like Growth Factor-2 mRNA Binding Proteins (IGF2BPs/IMPs/VICKZs) bind RNA	1.40E-04	2.44E-02	Reactome
12	Collagen biosynthesis and modifying enzymes	1.54E-04	2.45E-02	Reactome
13	PI3K-Akt signaling pathway	1.87E-04	2.48E-02	Wikipathways
14	SHP2 signaling	1.99E-04	2.48E-02	PID
15	MAPK Signaling Pathway	2.00E-04	2.48E-02	Wikipathways
16	Bladder cancer - Homo sapiens (human)	2.07E-04	2.48E-02	KEGG
17	Prolactin signaling pathway - Homo sapiens (human)	2.36E-04	2.66E-02	KEGG
18	Transcriptional Regulation by VENTX	2.94E-04	2.96E-02	Reactome
19	O-linked glycosylation	2.94E-04	2.96E-02	Reactome
20	MAPK signaling pathway - Homo sapiens (human)	3.27E-04	3.03E-02	KEGG
21	p53 signaling pathway - Homo sapiens (human)	3.54E-04	3.03E-02	KEGG
22	Integrins in angiogenesis	3.62E-04	3.03E-02	PID
23	nfat and hypertrophy of the heart	3.64E-04	3.03E-02	BioCarta

24	Proteoglycans in cancer - Homo sapiens (human)	4.24E-04	3.38E-02	KEGG
25	Axon guidance	4.63E-04	3.54E-02	Reactome
26	Focal Adhesion-PI3K-Akt-mTOR-signaling pathway	5.15E-04	3.54E-02	Wikipathways
27	Mechanoregulation and pathology of YAP-TAZ via Hippo and non-Hippo mechanisms	5.16E-04	3.54E-02	Wikipathways
28	Pancreatic cancer - Homo sapiens (human)	5.18E-04	3.54E-02	KEGG
29	mucin core 1 and core 2 O-glycosylation	5.72E-04	3.78E-02	HumanCyc
30	Other types of O-glycan biosynthesis - Homo sapiens (human)	6.09E-04	3.89E-02	KEGG
31	Nervous system development	7.11E-04	4.10E-02	Reactome
32	Signaling by FLT3 ITD and TKD mutants	7.29E-04	4.10E-02	Reactome
33	btg family proteins and cell cycle regulation	7.42E-04	4.10E-02	BioCarta
34	Small interfering RNA (siRNA) biogenesis	7.42E-04	4.10E-02	Reactome
35	Autophagy - animal - Homo sapiens (human)	7.50E-04	4.10E-02	KEGG
36	PI3K-Akt signaling pathway - Homo sapiens (human)	8.10E-04	4.31E-02	KEGG
37	Bladder cancer	9.01E-04	4.66E-02	Wikipathways
38	Gene expression (Transcription)	9.47E-04	4.77E-02	Reactome
39	Relaxin signaling pathway - Homo sapiens (human)	1.07E-03	5.00E-02	KEGG
40	Mucin type O-glycan biosynthesis - Homo sapiens (human)	1.08E-03	5.00E-02	KEGG
41	mTOR signaling pathway - Homo sapiens (human)	1.08E-03	5.00E-02	KEGG
42	Generic Transcription Pathway	1.10E-03	5.00E-02	Reactome
43	Signaling pathways regulating pluripotency of stem cells - Homo sapiens (human)	1.22E-03	5.27E-02	KEGG
44	Axon guidance - Homo sapiens (human)	1.27E-03	5.27E-02	KEGG
45	Neurotrophin signaling pathway - Homo sapiens (human)	1.28E-03	5.27E-02	KEGG
46	MECP2 regulates transcription factors	1.30E-03	5.27E-02	Reactome
47	Brain-derived neurotrophic factor (BDNF) signaling pathway	1.31E-03	5.27E-02	Wikipathways
48	TGF-beta Signaling Pathway	1.36E-03	5.27E-02	Wikipathways
49	Canonical and non-canonical TGF-B signaling	1.38E-03	5.27E-02	Wikipathways

50	Regulation of RUNX1 Expression and Activity	1.38E-03	5.27E-02	Reactome

B

Rank	***miR-148b-5p* mediated pathway**	p-value	q-value	pathway source
1	TGF-beta Receptor Signaling	5.78E-05	3.68E-02	Wikipathways
2	TGF-beta receptor signaling in skeletal dysplasias	9.66E-05	3.68E-02	Wikipathways
3	BMP Signaling Pathway	1.33E-04	3.68E-02	HumanCyc
4	RUNX3 regulates BCL2L11 (BIM) transcription	1.45E-04	3.68E-02	Reactome
5	Signaling by BMP	3.89E-04	6.88E-02	Reactome
6	Beta-catenin phosphorylation cascade	4.08E-04	6.88E-02	Reactome
7	Phase 1 - inactivation of fast Na+ channels	4.91E-04	6.92E-02	Reactome
8	XAV939 stabilizes AXIN	6.13E-04	6.92E-02	Reactome
9	Signaling by TGFB family members	6.19E-04	6.92E-02	Reactome
10	Circadian rhythm pathway	6.83E-04	6.92E-02	PID
11	Circadian rhythm - Homo sapiens (human)	9.08E-04	7.66E-02	KEGG
12	Tight junction interactions	9.08E-04	7.66E-02	Reactome
13	Sodium/Proton exchangers	1.13E-03	7.81E-02	Reactome
14	Platelet homeostasis	1.20E-03	7.81E-02	Reactome
15	Circadian Clock	1.22E-03	7.81E-02	Reactome
16	Transcriptional regulation by RUNX3	1.26E-03	7.81E-02	Reactome
17	Signaling by NODAL	1.31E-03	7.81E-02	Reactome
18	Regulation of MECP2 expression and activity	1.40E-03	7.87E-02	Reactome
19	CTLA4 inhibitory signaling	1.59E-03	8.46E-02	Reactome
20	Signaling by WNT in cancer	1.81E-03	9.15E-02	Reactome
21	Sudden Infant Death Syndrome (SIDS) Susceptibility Pathways	2.04E-03	9.83E-02	Wikipathways
22	Degradation of beta-catenin by the destruction complex	2.33E-03	1.07E-01	Reactome
23	ERKs are inactivated	2.81E-03	1.19E-01	Reactome

24	Signaling by Activin	2.81E-03	1.19E-01	Reactome
25	BMP receptor signaling	3.27E-03	1.30E-01	PID
26	Toxicity of botulinum toxin type A (botA)	3.56E-03	1.30E-01	Reactome
27	BMAL1:CLOCK,NPAS2 activates circadian gene expression	3.59E-03	1.30E-01	Reactome
28	Neural Crest Differentiation	3.59E-03	1.30E-01	Wikipathways
29	Integrin-linked kinase signaling	4.92E-03	1.68E-01	PID
30	Cell-cell junction organization	4.97E-03	1.68E-01	Reactome
31	Disassembly of the destruction complex and recruitment of AXIN to the membrane	5.40E-03	1.69E-01	Reactome
32	Regulation of Microtubule Cytoskeleton	5.41E-03	1.69E-01	Wikipathways
33	telomeres telomerase cellular aging and immortality	5.50E-03	1.69E-01	BioCarta
34	RUNX3 regulates CDKN1A transcription	5.83E-03	1.69E-01	Reactome
35	RUNX3 Regulates Immune Response and Cell Migration	5.83E-03	1.69E-01	Reactome
36	Cell junction organization	6.55E-03	1.84E-01	Reactome
37	wnt signaling pathway	6.88E-03	1.88E-01	BioCarta
38	Autophagy - animal - Homo sapiens (human)	7.09E-03	1.89E-01	KEGG
39	TGF-beta super family signaling pathway canonical	7.81E-03	2.01E-01	INOH
40	Platelet sensitization by LDL	7.93E-03	2.01E-01	Reactome
41	IL-6 signaling	8.60E-03	2.02E-01	INOH
42	Synthesis of wybutosine at G37 of tRNA(Phe)	8.60E-03	2.02E-01	Reactome
43	AndrogenReceptor	8.89E-03	2.02E-01	NetPath
44	Transcription co-factors SKI and SKIL protein partners	9.34E-03	2.02E-01	Wikipathways
45	IL8- and CXCR2-mediated signaling events	9.57E-03	2.02E-01	PID
46	BMP signaling Dro	9.57E-03	2.02E-01	INOH
47	RAF activation	9.57E-03	2.02E-01	Reactome
48	RHOU GTPase cycle	9.57E-03	2.02E-01	Reactome
49	Synthesis of PIPs at the plasma membrane	9.85E-03	2.03E-01	Reactome

50	PP2A-mediated dephosphorylation of key metabolic factors	1.18E-02	2.36E-01	Reactome

C

Rank	*miR-146a-5p* mediated pathway	p-value	q-value	pathway source
1	Herpes simplex virus 1 infection - Homo sapiens (human)	8.73E-05	8.55E-02	KEGG
2	Generic Transcription Pathway	2.59E-04	1.01E-01	Reactome
3	Gene expression (Transcription)	4.46E-04	1.01E-01	Reactome
4	RNA Polymerase II Transcription	4.59E-04	1.01E-01	Reactome
5	NOTCH-Ncore	5.14E-04	1.01E-01	Signalink
6	MECP2 regulates neuronal receptors and channels	8.05E-04	1.31E-01	Reactome
7	TCR signaling in naïve CD4+ T cells	1.18E-03	1.65E-01	PID
8	Wnt	1.41E-03	1.72E-01	NetPath
9	RAC1 signaling pathway	1.92E-03	2.09E-01	PID
10	Neuronal System	3.38E-03	2.40E-01	Reactome
11	Transport of inorganic cations/anions and amino acids/oligopeptides	3.42E-03	2.40E-01	Reactome
12	NGF processing	3.43E-03	2.40E-01	Reactome
13	Expression and Processing of Neurotrophins	3.43E-03	2.40E-01	Reactome
14	miR-222 in Exercise-Induced Cardiac Growth	3.43E-03	2.40E-01	Wikipathways
15	Membrane Trafficking	3.94E-03	2.50E-01	Reactome
16	Mesodermal commitment pathway	4.24E-03	2.50E-01	Wikipathways
17	Transcriptional Regulation by MECP2	4.55E-03	2.50E-01	Reactome
18	Neddylation	5.22E-03	2.50E-01	Reactome
19	H19 action Rb-E2F1 signaling and CDK-Beta-catenin activity	5.23E-03	2.50E-01	Wikipathways
20	Nucleobase biosynthesis	5.23E-03	2.50E-01	Reactome
21	Protein-protein interactions at synapses	5.59E-03	2.50E-01	Reactome
22	Activation of NOXA and translocation to mitochondria	5.63E-03	2.50E-01	Reactome
23	CD28 co-stimulation	7.24E-03	2.95E-01	Reactome

24	Amino acid transport across the plasma membrane	7.24E-03	2.95E-01	Reactome
25	Glycogen breakdown (glycogenolysis)	7.54E-03	2.95E-01	Reactome
26	Vesicle-mediated transport	8.83E-03	3.20E-01	Reactome
27	Fc gamma R-mediated phagocytosis - Homo sapiens (human)	8.91E-03	3.20E-01	KEGG
28	Posttranslational regulation of adherens junction stability and dissassembly	9.15E-03	3.20E-01	PID
29	TCR signaling in naïve CD8+ T cells	1.07E-02	3.34E-01	PID
30	TRAF6 mediated IRF7 activation in TLR7/8 or 9 signaling	1.14E-02	3.34E-01	Reactome
31	Wnt signaling pathway and pluripotency	1.16E-02	3.34E-01	Wikipathways
32	Signaling by Hippo	1.20E-02	3.34E-01	Reactome
33	PDGFR-beta signaling pathway	1.24E-02	3.34E-01	PID
34	CTLA4 inhibitory signaling	1.37E-02	3.34E-01	Reactome
35	Cargo recognition for clathrin-mediated endocytosis	1.42E-02	3.34E-01	Reactome
36	Viral myocarditis - Homo sapiens (human)	1.42E-02	3.34E-01	KEGG
37	Signaling events regulated by Ret tyrosine kinase	1.45E-02	3.34E-01	PID
38	Regulation of mRNA stability by proteins that bind AU-rich elements	1.45E-02	3.34E-01	Reactome
39	Signal Transduction	1.47E-02	3.34E-01	Reactome
40	Role of ABL in ROBO-SLIT signaling	1.50E-02	3.34E-01	Reactome
41	ARMS-mediated activation	1.50E-02	3.34E-01	Reactome
42	Inactivation of CDC42 and RAC1	1.50E-02	3.34E-01	Reactome
43	HuR (ELAVL1) binds and stabilizes mRNA	1.50E-02	3.34E-01	Reactome
44	EPH-ephrin mediated repulsion of cells	1.50E-02	3.34E-01	Reactome
45	PI3K-AKT-mTOR - VitD3 signaling	1.56E-02	3.40E-01	Wikipathways
46	Ebstein-Barr virus LMP1 signaling	1.76E-02	3.45E-01	Wikipathways
47	how progesterone initiates the oocyte maturation	1.76E-02	3.45E-01	BioCarta
48	Glutamate Neurotransmitter Release Cycle	1.76E-02	3.45E-01	Reactome
49	Metabolic reprogramming in colon cancer	1.87E-02	3.45E-01	Wikipathways

50	p75NTR recruits signaling complexes	1.90E-02	3.45E-01	Reactome

Supplementary Table 6. Prediction results of honeysuckle- and Huangqi-induced miRNA.

**TCM**	**Prediction (adjusted *p* value)***		
	hsa-let7a-5p	***hsa-miR148b-5p***	***hsa-miR-146a-5p***
**Honeysuckle**	6.241×10^{-8}	2.368×10^{-2}	2.385×10^{-11}
**Huangqi**	6.488×10^{-10}	not predicted	3.589×10^{-11}

* Prediction parameters:

SymMap: targets of TCM are filtered with FDR-BH < 0.05.

g:Profiler: statistical domain scope is set as "all known genes"; significance threshold is set as FDR-BH < 0.05.
